# Supplementary material for: Social identity correlates of social media engagement before and after the 2022 Russian invasion of Ukraine
Source: Nat Commun. 2024 Oct 1;15:8127. doi: 10.1038/s41467-024-52179-8 (PMC11445580; doi:10.1038/s41467-024-52179-8)
Supplement: Supplementary file 1 — Supplementary Information [file 41467_2024_52179_MOESM1_ESM.pdf]

Supplementary Information

Social identity correlates of social media engagement before and after the 2022 Russian invasion of Ukraine

Yara Kyrychenko, Tymofii Brik, Sander van der Linden, Jon Roozenbeek

Email: yk408@cam.ac.uk, jjr51@cam.ac.uk

Includes: Supplementary Methods, Supplementary Figures 1 to 10, Supplementary Tables 1 to 31.

Table of Contents

1. **Supplementary Methods** 3

2. **Supplementary Figures** 4

2.1. Supplementary Fig. 1. Engagement (reactions) with pro-Ukrainian news sources content before the invasion. 4

2.2. Supplementary Fig. 2. Time series of exp(estimate) for pro-Ukrainian Twitter (sliding window of 14 days). 5

2.3. Supplementary Fig. 3. Word clouds of top 200 words from the ingroup (A) and outgroup (B) mentions dictionaries on Facebook. 6

2.4. Supplementary Fig. 4. Word clouds of top 200 words from the in-group solidarity (A) and out-group hostility (B) dictionaries on pro-Ukrainian Facebook. 6

2.5. Supplementary Fig. 5. Time series of exp(estimate) for pro-Ukrainian Facebook. Sliding window of 21 days. 7

2.6. Supplementary Fig. 6. Time series of exp(estimate) for pro-Ukrainian Facebook predicting log-transformed engagement based on the count of words in the dictionaries of ingroup solidarity, outgroup hostility, ingroup and outgroup mentions. 8

2.7. Supplementary Fig. 7. Time series of exp(estimate) for pro-Ukrainian Twitter predicting log-transformed engagement based on the count of words in the dictionaries of ingroup solidarity, outgroup hostility, ingroup and outgroup mentions. 9

2.8. Supplementary Fig. 8. Time series of exp(estimate) for pro-Ukrainian Facebook predicting log(Love +1) (sliding window of 14 days). 10

2.9. Supplementary Fig. 9. Time series of exp(estimate) for pro-Ukrainian Facebook predicting log(Angry +1) (sliding window of 14 days). 11

2.10. Supplementary Fig. 10. Distributions of posts and the proportion of posts classified as ingroup solidarity, outgroup hostility, and binary ingroup and outgroup mentions over time on (A) pro-Ukrainian Facebook news sources, (B) pro-Ukrainian Twitter news sources, (C) pro-Ukrainian Twitter geolocated. 12

3. **Supplementary Tables** 13

3.1. Supplementary Table 1. Study 1: Predictors of engagement on Ukrainian social media before the invasion. 13

3.2. Supplementary Table 2. Study 2: Predictors of engagement on Ukrainian social media after the invasion. 14

3.3. Supplementary Table 3. Study 2: Predictors of engagement on Ukrainian social media with ingroup solidarity and outgroup hostility. 15

3.4. Supplementary Table 4. Study 3: Predictors of engagement on geolocated Ukrainian Twitter before and after the invasion. 16

3.5. Supplementary Table 5. Descriptive Statistics for pro-Ukrainian news sources on Facebook before and after the invasion. 17

3.6. Supplementary Table 6. Descriptive Statistics for pro-Ukrainian news sources on Twitter before and after the invasion. 17

3.7. Supplementary Table 7. Descriptive Statistics for pro-Russian news sources on Facebook and Twitter (before the invasion). 18

3.8. Supplementary Table 8. Descriptive Statistics for pro-Ukrainian geolocated Twitter before and after the invasion. 18

3.9. Supplementary Table 9. Correlation matrix for pro-Ukrainian news sources on Facebook. 19

3.10. Supplementary Table 10. Correlation matrix for pro-Ukrainian news sources on Twitter. 19

3.11. Supplementary Table 11. Correlation matrix for geolocated pro-Ukrainian Twitter. 19

3.12. Supplementary Table 12. Descriptive statistics of Facebook reactions for pro-Ukrainian news sources before the invasion. 20

3.13. Supplementary Table 13. Descriptive statistics of Facebook reactions for pro-Ukrainian news sources after the invasion. 20

3.14. Supplementary Table 14. Descriptive statistics of Facebook reactions for pro-Russian news sources before the invasion. 20

3.15. Supplementary Table 15. Classifier validation results (Facebook and Twitter news sources). Results are based on a dataset of 400 posts stratified by class (different for ingroup solidarity and outgroup hostility). 21

3.16. Supplementary Table 16. Classifier validation results (geolocated Twitter). Results for everything but pro-Ukrainian classifier are based on the data classified as pro-Ukrainian by the model (309 posts). 21

3.17. Supplementary Table 17. Study 1: Predictors of Twitter reactions (RT and Favorite) on Ukrainian social media before and after the invasion. 22

3.18. Supplementary Table 18. Study 1: Predictors of Facebook reactions on Ukrainian social media before the invasion (pro-Ukrainian). 24

3.19. Supplementary Table 19. Study 1: Predictors of Facebook reactions on Ukrainian social media before the invasion (pro-Russian). 25

3.20. Supplementary Table 20. Study 1: Predictors of Facebook reactions on Ukrainian social media after the invasion. 26

3.21. Supplementary Table 21. Predictors of engagement on Ukrainian social media after the invasion without identity mentions variables. 27

3.22. Supplementary Table 22. Study 3: Predictors of engagement on geolocated Ukrainian Twitter before and after the invasion (only posts where full user data is available). 28

3.23. Supplementary Table 23. Study 1: Predictors of engagement on geolocated Ukrainian Twitter before and after the invasion. 29

3.24. Supplementary Table 24. Study 1 Variance Inflation Factors. 30

3.25. Supplementary Table 25. Study 2: Predictors of Twitter reactions on Ukrainian social media before and after the invasion. 31

3.26. Supplementary Table 26. Study 2: Predictors of Facebook reactions on Ukrainian social media before the invasion. 32

3.27. Supplementary Table 27. Study 2: Predictors of Facebook reactions on Ukrainian social media after the invasion. 33

3.28. Supplementary Table 28. Study 2 Variance Inflation Factors. 34

3.29. Supplementary Table 29. Study 3 Variance Inflation Factors. 35

3.30. Supplementary Table 30. Study 3: Predictors of engagement on geolocated Ukrainian Twitter before and after the invasion for BERT-NLI classification without the .999 probability threshold do not change. 36

3.31. Supplementary Table 31. Study 2: Predictors of engagement on Ukrainian social media after the invasion with the dictionary approach. 37

1. Supplementary Methods

News sources outlet selection. We selected the most popular news sources in Ukraine based on the following sources: the Internet Association of Ukraine 100 Most Popular Socio-Political Websites in Ukraine (Dec 2019) (https://inau.ua/news/novyny-inau/top-100-novynnykh-saytiv-suspilno-politychnoyi-tematyky-za-hruden-2019), the Institute of Mass Information Rating of Ukraine Websites (Aug 2020) (https://imi.org.ua/en/monitorings/rating-of-ukraine-websites-i35051), Texty.org.ua 50 Most Read Online News Media based on Gemius and TNS rankings (https://texty.org.ua/d/2018/media-ranking/list.html), VoxUkraine Media Experts Study (https://voxukraine.org/ti-hto-poyasnyuye-svit-kogo-media-vvazhayut-ekspertamy/), and SimilarWeb Top 15 (Jul-Sept 2021) (https://www.similarweb.com). In total, we compiled a list of 108 media of which only 102 had either a Facebook or Twitter account that were functioning as of August 2021.

News sources outlet classification. We classified news sources as pro-Russian if, in August 2021, they were based in Russia or banned in Ukraine for being pro-Russian (ban on strana.ua: https://www.rferl.org/a/ukraine-sanctions-russia-derkach/31421149.html, ban on ZIK, NewsOne, and 112 Ukraine: https://www.dw.com/en/ukraine-zelenskiy-bans-three-opposition-tv-stations/a-56438505). For the banned websites, social media like Facebook, Twitter and YouTube became the primary means of distribution. All other news sources were classified as pro-Ukrainian (see OSF 10.17605/OSF.IO/RMC3E for the list of news sources and classifications at lists/UkraineMediaList.csv).

Control variables. Every tweet in our sample contained media and URL, so “has media” and “has URL” were not included in the control variables for Twitter. For Twitter, we controlled for “total tokens”, “follower count”, “is retweet” (not in Study 2 because the variable only had one factor for many sliding windows), while for Facebook we controlled for “total tokens”, “followers at posting”,“has media”, “has URL.” For Study 3, we controlled for “total tokens”, “follower count”, “has URL”, and “verified,” which could be either True, False or NA, indicating missing user data. These variables were scaled: “total tokens”, “follower count”, “followers at posting”.

Linear mixed models normality and equal variances assumptions. We chose linear mixed models to account for the non-independence of the posts given that multiple of them can come from the same source. For the main models, the check for normality and equal variances can be found on OSF (10.17605/OSF.IO/RMC3E) under plots/diagnostic. Some slight deviations from normality are visible in the QQ plots, as expected of real world data sets. The fitted versus residual plots show mostly expected patterns given the dependent variable is bounded below, with the exception of pro-Russian Twitter before invasion. We conducted robust linear mixed models using robustlmm R package for the main models and found similar results.

Descriptive statistics. Descriptive statistics for the datasets can be found in Tables S5-S8. The correlation matrices for the language constructs in the datasets are in Tables S9-S11. Tables S12-S14 have the correlations and means and standard deviations for Facebook reactions.

Dictionary and model performance. F1-macro, balanced accuracy and accuracy for the dictionaries and models are in Tables S15 and S16.

Ingroup mentions dictionary (translated, the original dictionaries include the appropriate morphological derivatives):
Ukraine, Kyiv, Kiev, Kharkiv, Lviv, Lvov, Zaporizhzhia, Odessa, Dnipro, Mykolaiv, Vinnytsia, Kryvyi Rih, Zhytomyr, Ivano-Frankivsk, Sumy, Hryvna, Bankova, Zelenskyy, Avakov, Yermak, Medvedchuk, Poroshenko, Shefir. Venediktov, Shmygal. Razumkov, Kosiuk.

Outgroup mentions dictionary (translated, the original dictionaries include the appropriate morphological derivatives):
Russia, RF, Moscow, Saint Petersburg, Petersburg, Peter, Novosibirsk, Ekaterinburg, Samara, Kazan, Omsk, Chelyabinsk, Rostov, Ufa, Novgorod, Volgograd, Krasnoyarsk, Perm, Voronezh, Khabarovsk, Pyatigorsk, Ruble, Kremlin, Putin, Mishustin, Vaimo, Medvedev, Shoigu, Kiriyenko, Sobyanin, Sechin, Lavrov, Miller.

Ingroup solidarity dictionary (translated, the original dictionaries include the appropriate morphological derivatives):
Glory, defender*, hero*, fighter*, battle*, light*, eternal, memory, we stand, defend*, defense, native, own land, homeland, warrior*, anthem, vyshyvanka , coat of arms, flag*, sich , 2014, viburnum*, mother, cossack*, spirit, volunteer*, legend*, perish*, cherish*, care*, protect*, victory, veteran*, cyborg*, borsch, palyanytsya, heavenly hundred, maidan.

Outgroup hostility dictionary (translated, the original dictionaries include the appropriate morphological derivatives):
Moskal*, katsap*, vatnik*, traitor*, fascist*, occupant*, occupier*, hostile, enemy, enemies, killer*, bully*, executioner*, rapist*, raped, scum, orc*, fool*, nonhuman*, revenge, punish, rashist*, invader*, backward, uncultured, cage, beast*, cockroach*, cold-blooded*, dominate, addict*, sick*, rude, creature, exterminate, extinct, wild, greedy, illogical, immoral, irrational, contagious, infection, irresponsible*, lazy, monkey*, monster*, neanderthal*, parasite*, pig*, poison*, predator*, mad*, rat*, outlaw, outcasts, spineless, subhuman, ignorant, illiterate, cancer, uncultured, undeveloped, ungrateful, stupid, venom*, pest*, vermin*, nest*, flea*, primitive*, bastard*, stranger*, alien*, blindly, sneaky, heartless, ruthless, materialistic, rob*, thief*, marauder*, passive, tough*, unsophisticated, unemotional, unreliable.

Dictionaries and code availability. All dictionaries (except LIWC) and code needed to replicate the analysis and train the classifiers in this paper are available on OSF: 10.17605/OSF.IO/RMC3E. Text of individual posts was not shared for privacy reasons. Please contact the corresponding author for full social media data.

Code book. The code book used in data labelling is on OSF: 10.17605/OSF.IO/RMC3E.

Robustness to variations in group mentions dictionaries. Our results are robust to dictionary changes. For instance, removing all cities or leaving only the country names in the dictionaries leads largely the same results (Table S23).

Variance Inflation Factors. All group identity VIFs were less than 1.4 in Study 1, the overall regressions from Study 2, and Study 3, indicating an absence of multicollinearity (Tables S24 and S28, S29). The VIFs for “has media” and “has URL” are higher because those two are very highly correlated in our data.

Robustness to variations in sliding window length. Our results do not change substantially for window sizes of 14 or 21 days (see Fig. S5). We chose a window of 14 days because it accounts for two full weekly news cycles, reducing the effects of weekday-weekend fluctuations in social media use, while being narrow enough to capture variations in engagement patterns.

2. Supplementary Figures

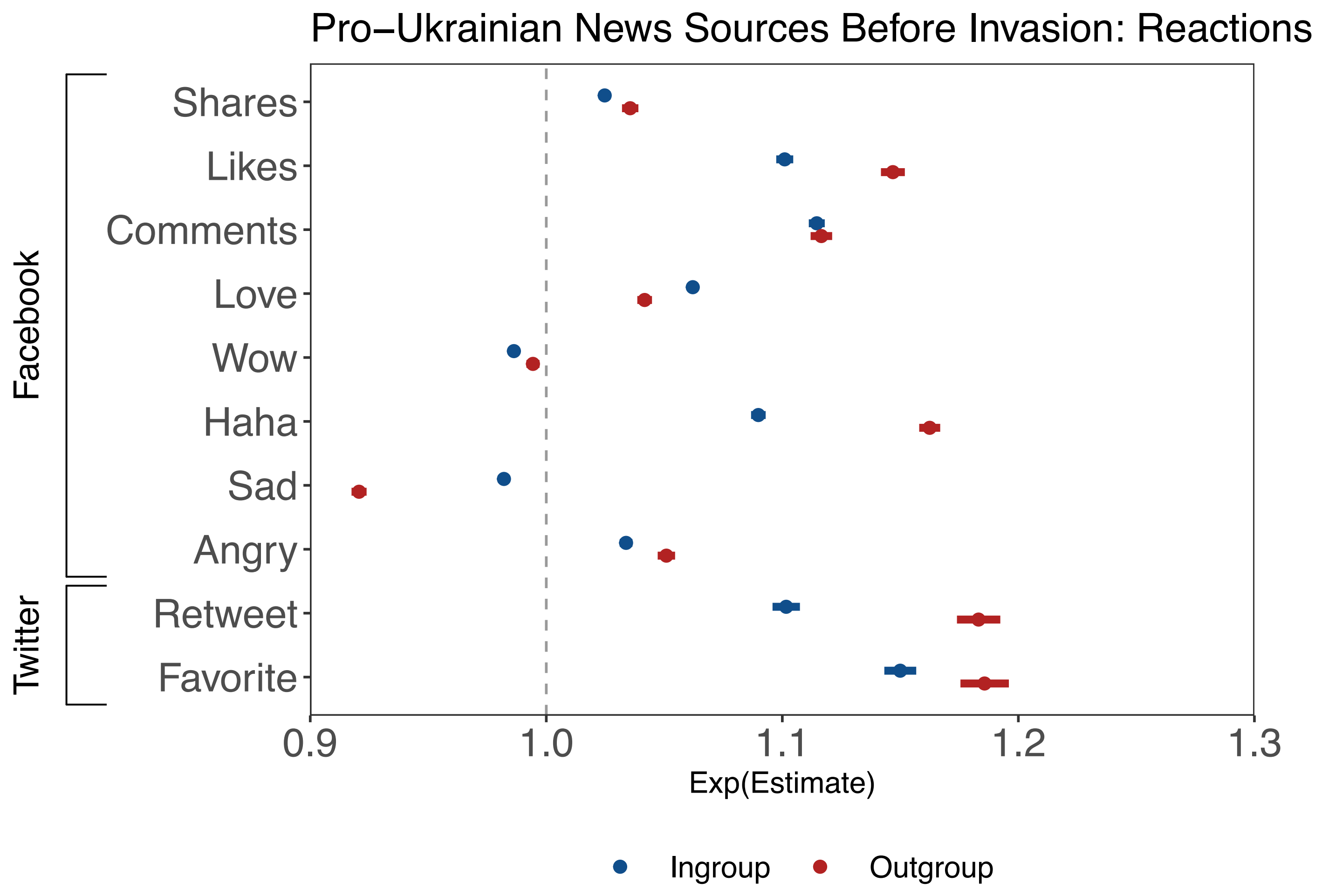

2.1. Supplementary Fig. 1. Engagement (reactions) with pro-Ukrainian news sources content before the invasion. Based on N=535,797 Facebook posts and N=217,245 tweets. The dots represent exp(estimate) and the bars represent 95%CI.

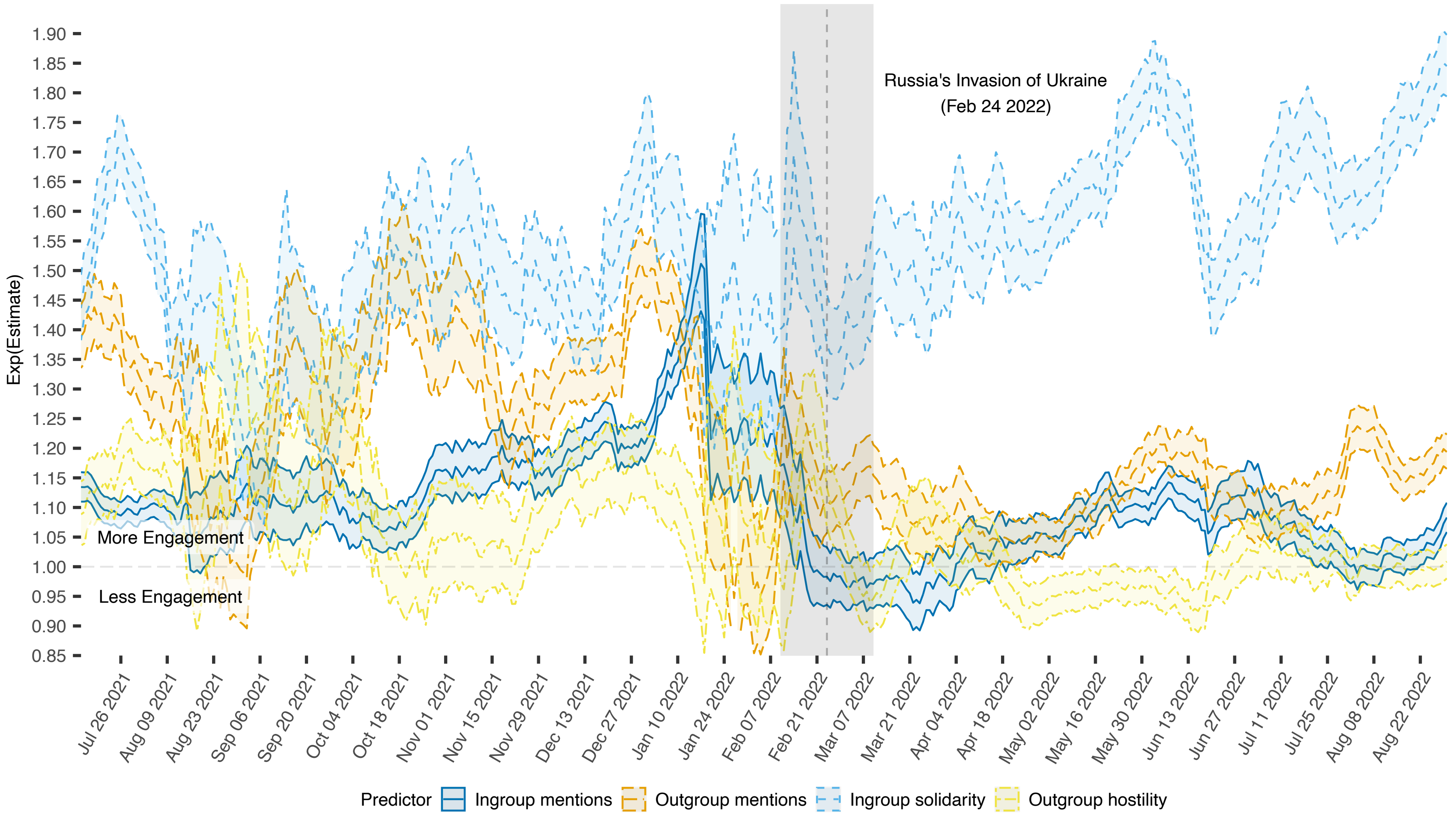

2.2. Supplementary Fig. 2. Time series of exp(estimate) for pro-Ukrainian Twitter (sliding window of 14 days).
Based on N = 399,555 Twitter posts. The dashed vertical line represents the invasion date (February 24, 2022), and the content from that day is present in the gray window around it. Data presented as time series of exp(estimate) (central lines) with 95% CIs (shaded regions around the central line); N = 1, 011, 171 Facebook posts.

A B

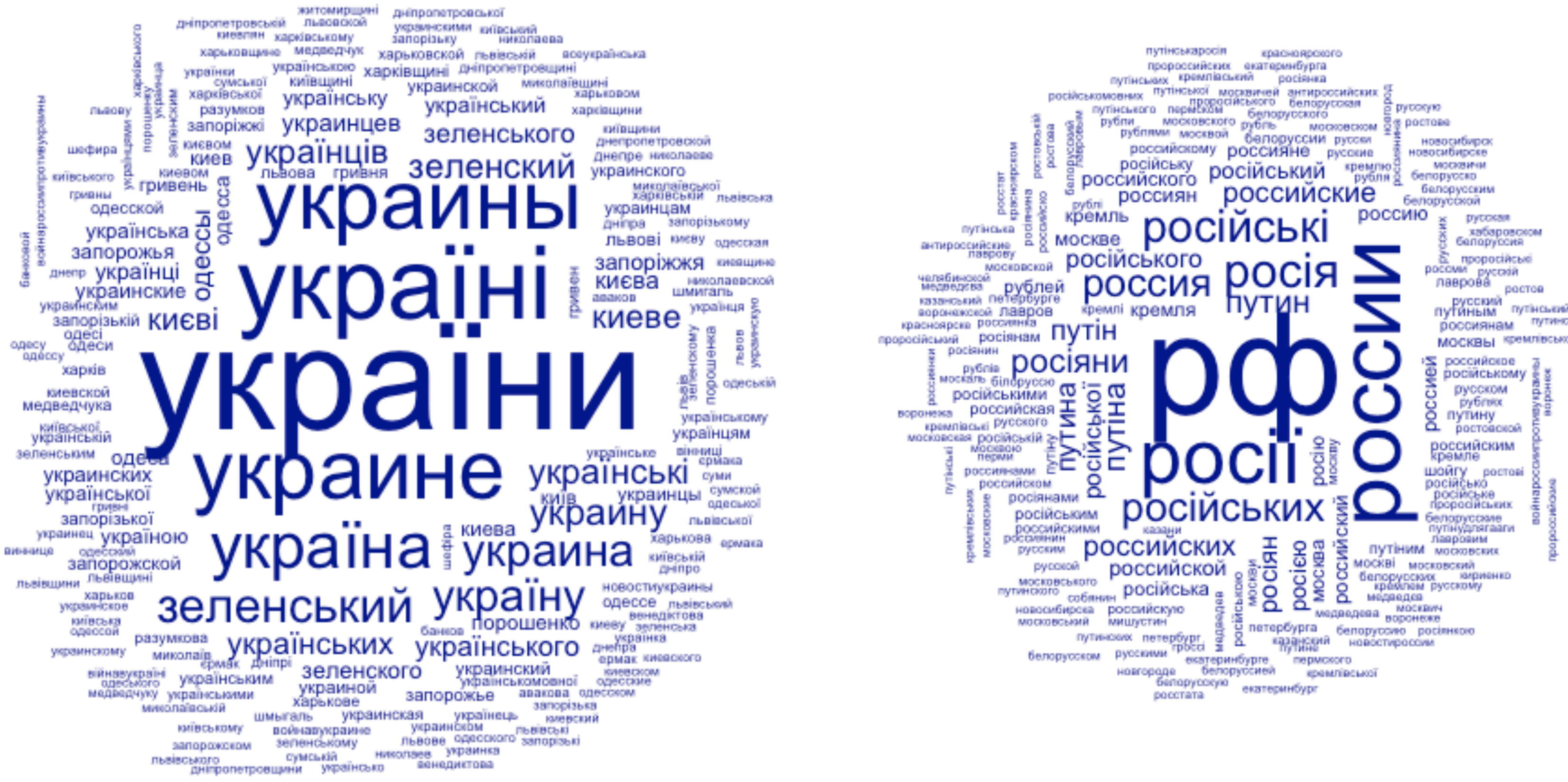

2.3. Supplementary Fig. 3. Word clouds of top 200 words from the ingroup (A) and outgroup (B) mentions dictionaries on Facebook.

The largest words in (A) are variations of the word Ukraine, while the largest words in (B) are variations of Russia and Russian.

A B

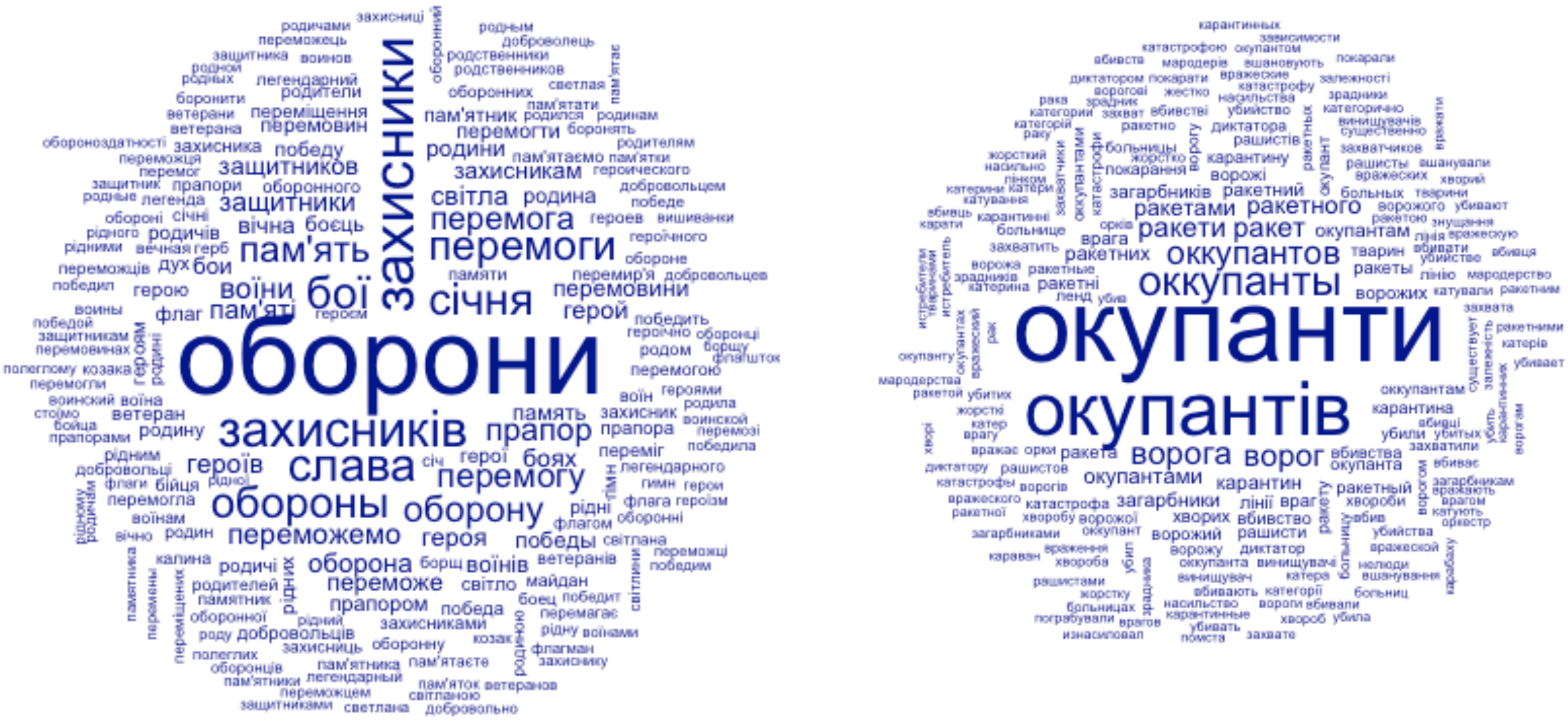

2.4. Supplementary Fig. 4. Word clouds of top 200 words from the in-group solidarity (A) and out-group hostility (B) dictionaries on pro-Ukrainian Facebook.

The largest words in (A) are (of) defense, defenders, and (of) defenders, as well as variations of glory, hero and victory, while the largest words in (B) are variations of occupiers and (of) occupiers, as well as rockets and enemy.

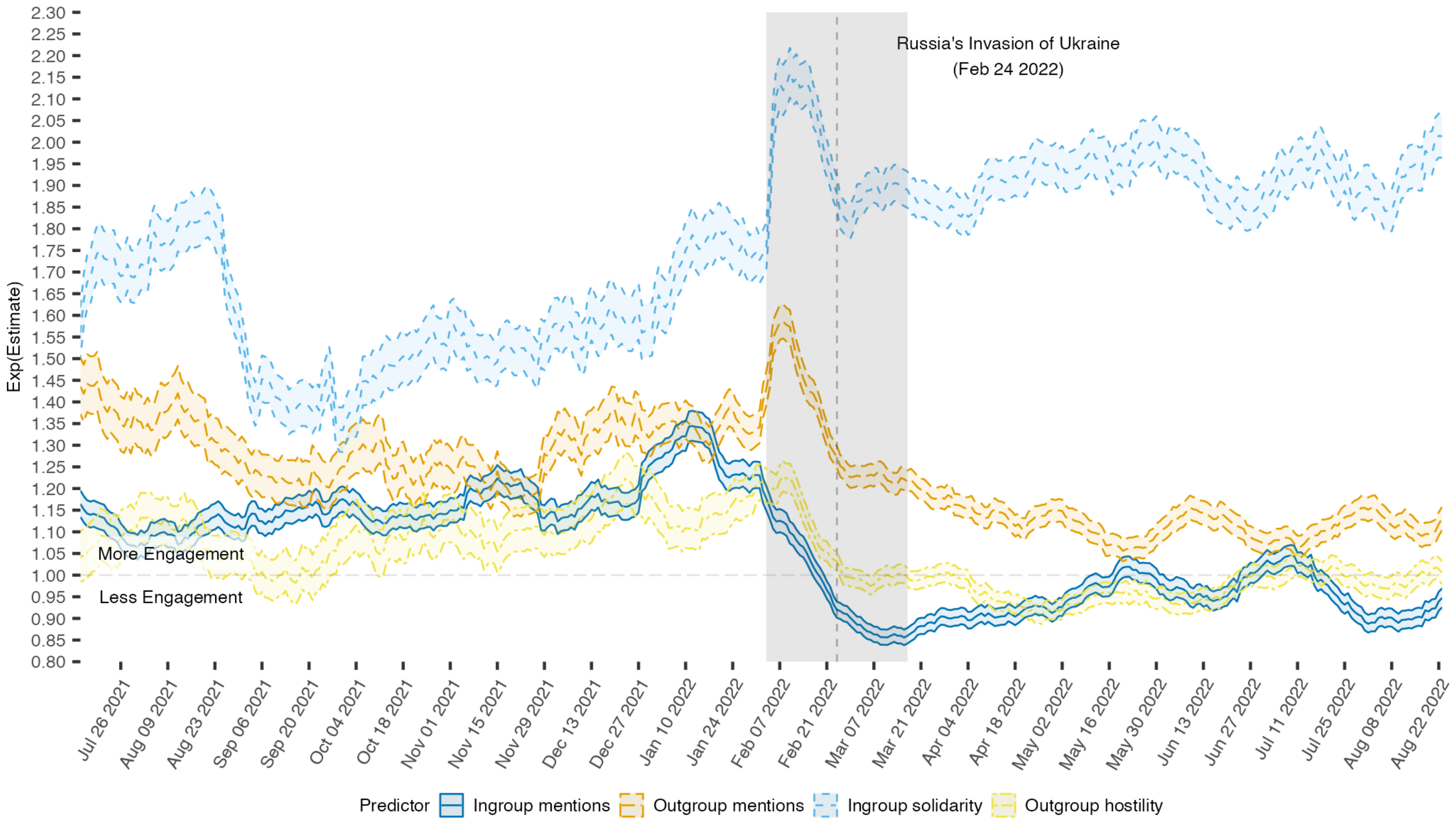

2.5. Supplementary Fig. 5. Time series of exp(estimate) for pro-Ukrainian Facebook. Sliding window of 21 days.
Based on N = 1, 011, 171 Facebook posts. The dashed vertical line represents the invasion date (February 24, 2022), and the content from that day is present in the gray window around it. Data presented as time series of exp(estimate) (central lines) with 95% CIs (shaded regions around the central line).

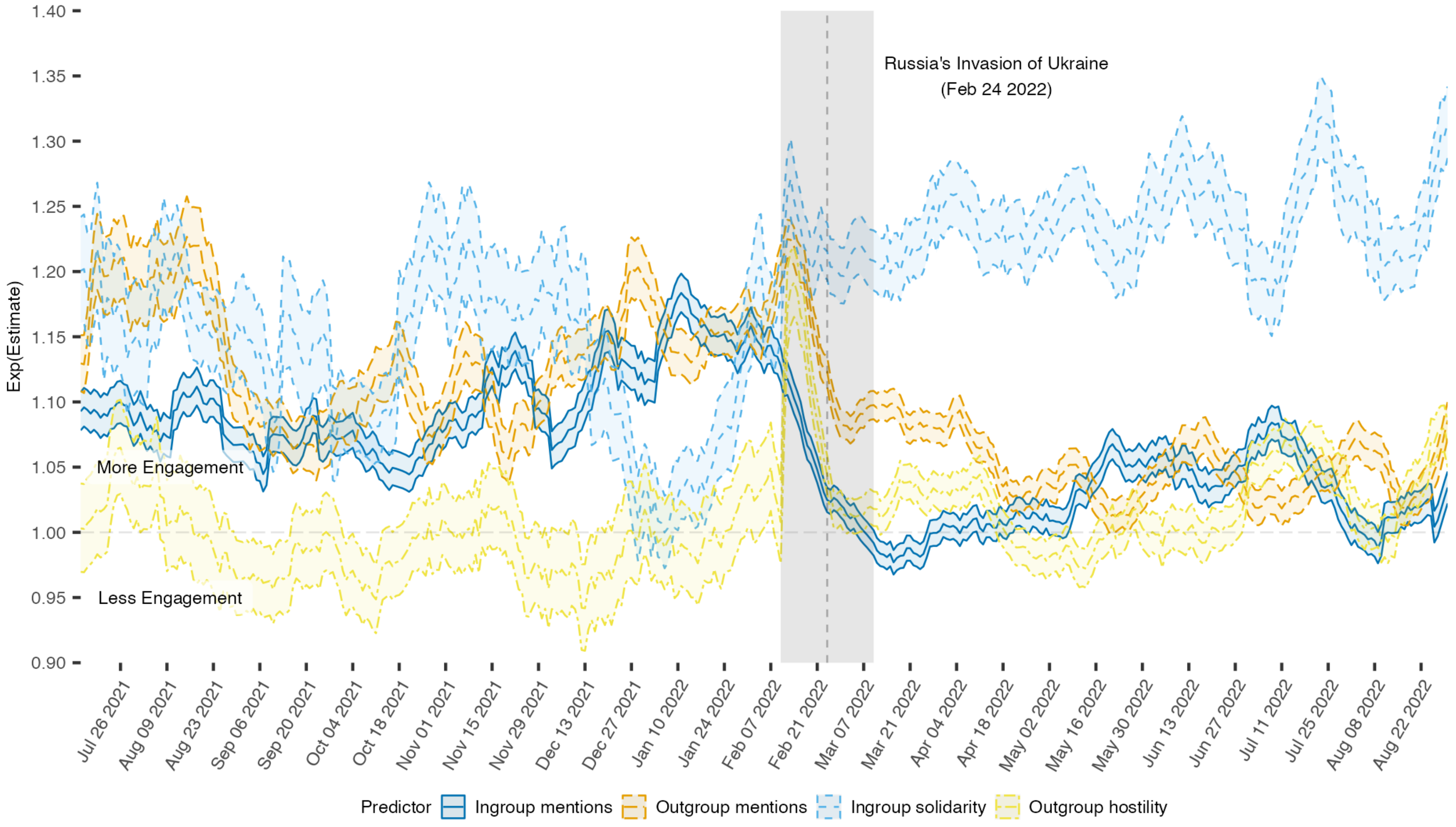

2.6. Supplementary Fig. 6. Time series of exp(estimate) for pro-Ukrainian Facebook predicting log-transformed engagement based on the count of words in the dictionaries of ingroup solidarity, outgroup hostility, ingroup and outgroup mentions. Based on N = 1, 011, 171 Facebook posts. The dashed vertical line represents the invasion date (February 24, 2022), and the content from that day is present in the gray window around it. Data presented as time series of exp(estimate) (central lines) with 95% CIs (shaded regions around the central line).

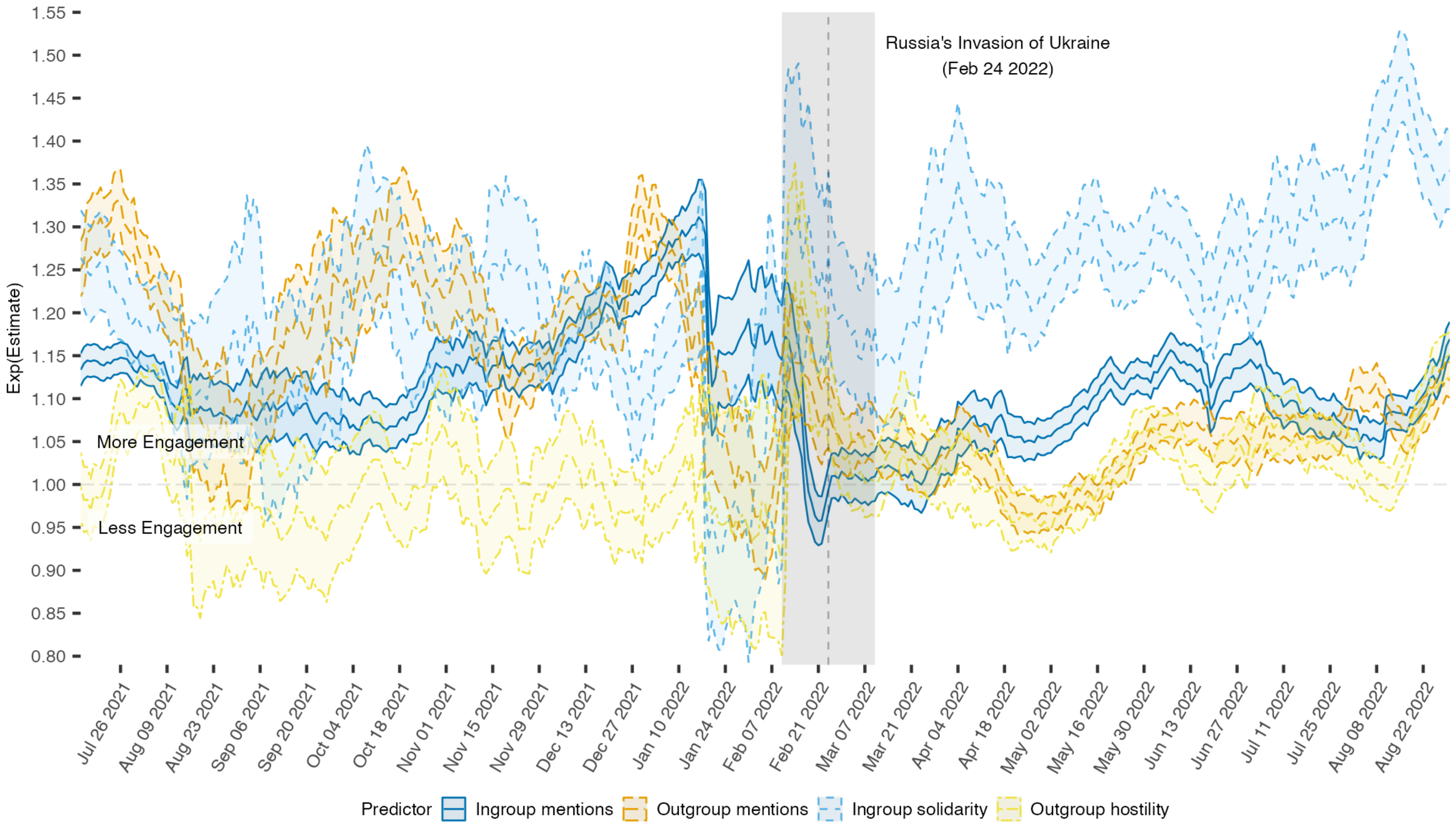

2.7. Supplementary Fig. 7. Time series of exp(estimate) for pro-Ukrainian Twitter predicting log-transformed engagement based on the count of words in the dictionaries of ingroup solidarity, outgroup hostility, ingroup and outgroup mentions. Based on N = 399,555 Twitter posts. The dashed vertical line represents the invasion date (February 24, 2022), and the content from that day is present in the gray window around it. Data presented as time series of exp(estimate) (central lines) with 95% CIs (shaded regions around the central line).

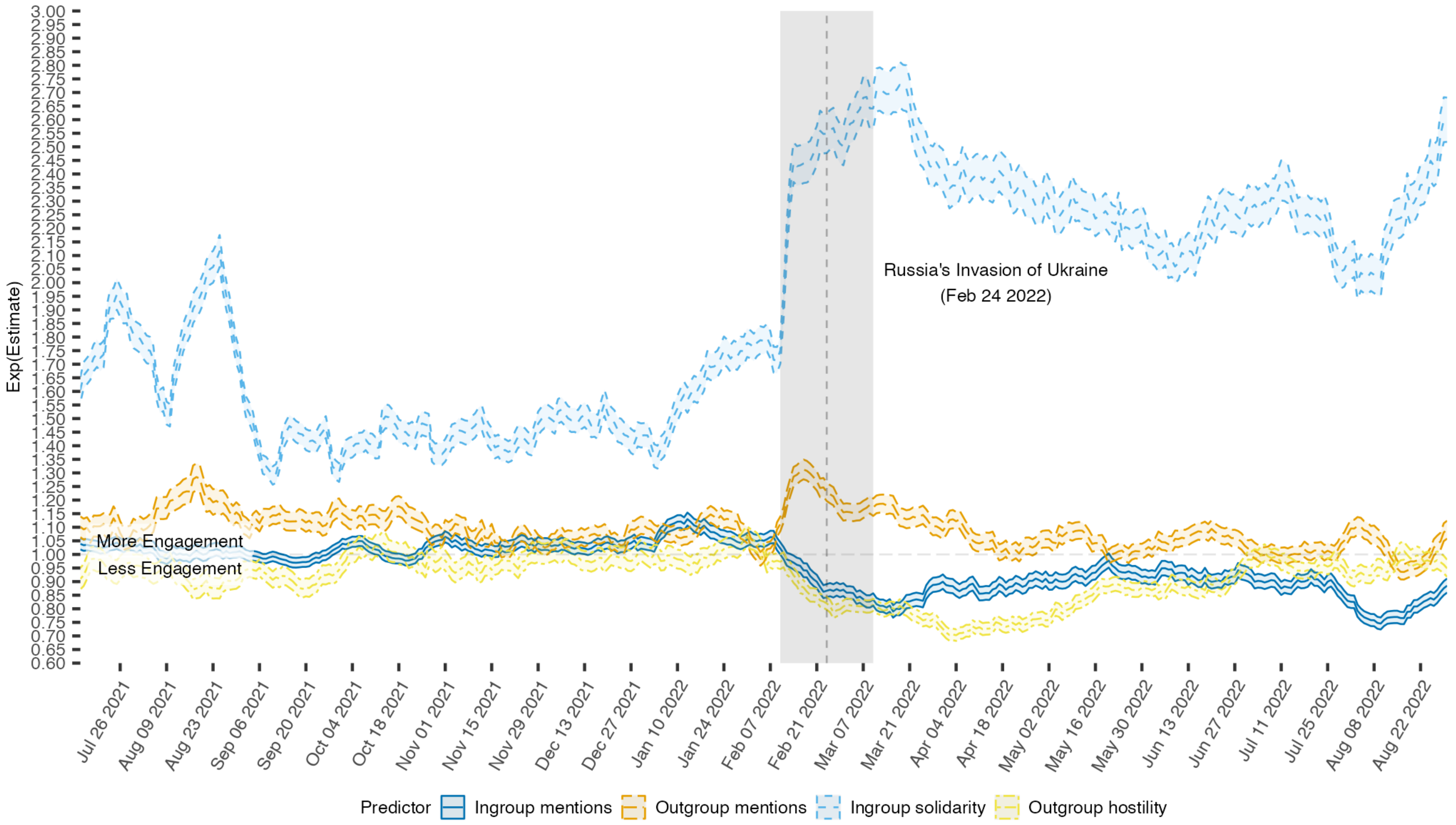

2.8. Supplementary Fig. 8. Time series of exp(estimate) for pro-Ukrainian Facebook predicting log(Love +1) (sliding window of 14 days).

Based on N = 1, 011, 171 Facebook posts. The dashed vertical line represents the invasion date (February 24, 2022), and the content from that day is present in the gray window around it. Data presented as time series of exp(estimate) (central lines) with 95% CIs (shaded regions around the central line).

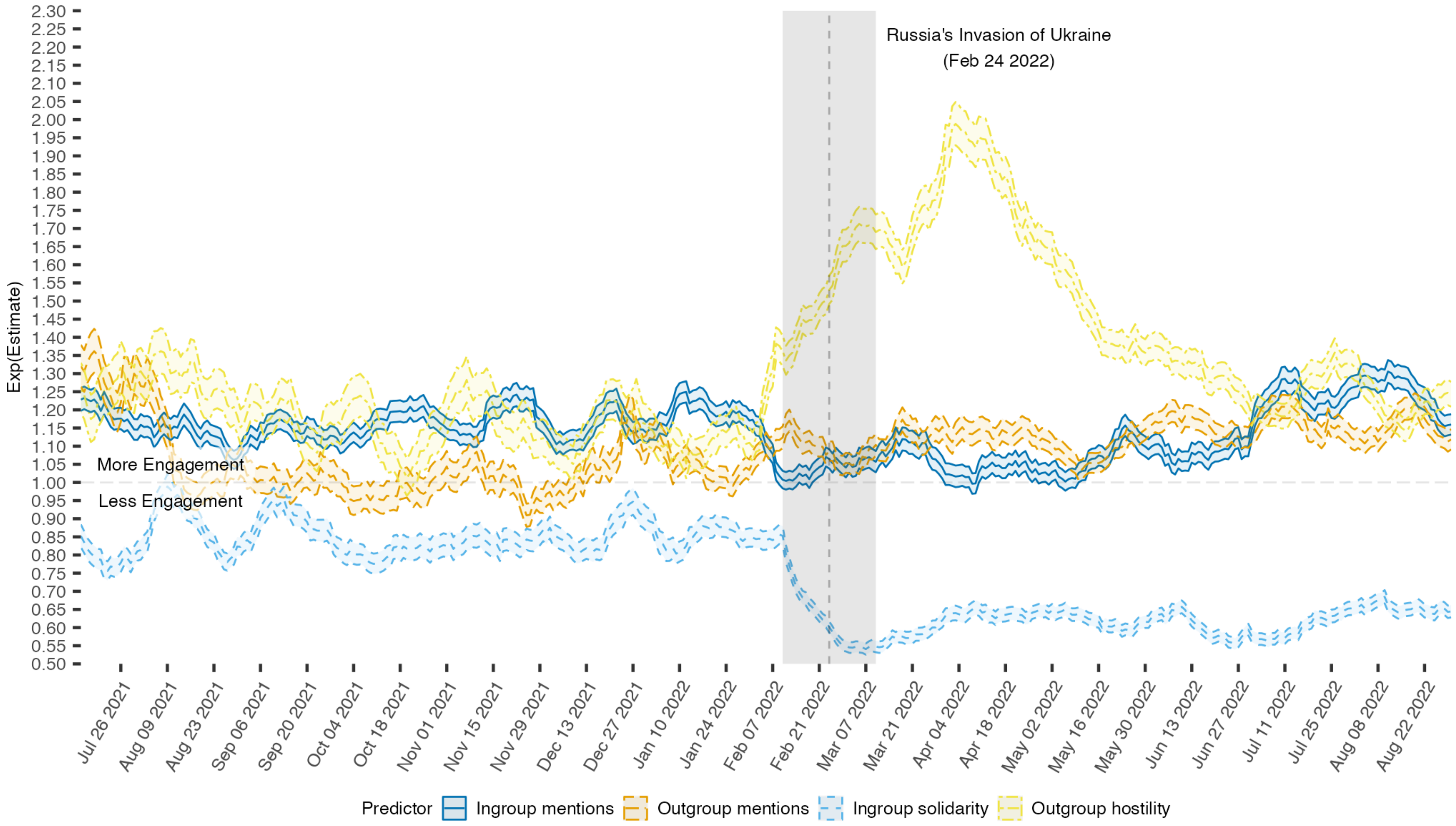

2.9. Supplementary Fig. 9. Time series of exp(estimate) for pro-Ukrainian Facebook predicting log(Angry +1) (sliding window of 14 days).

The dashed vertical line represents the invasion date (February 24, 2022), and the content from that day is present in the gray window around it. Data presented as time series of exp(estimate) (central lines) with 95% CIs (shaded regions around the central line).

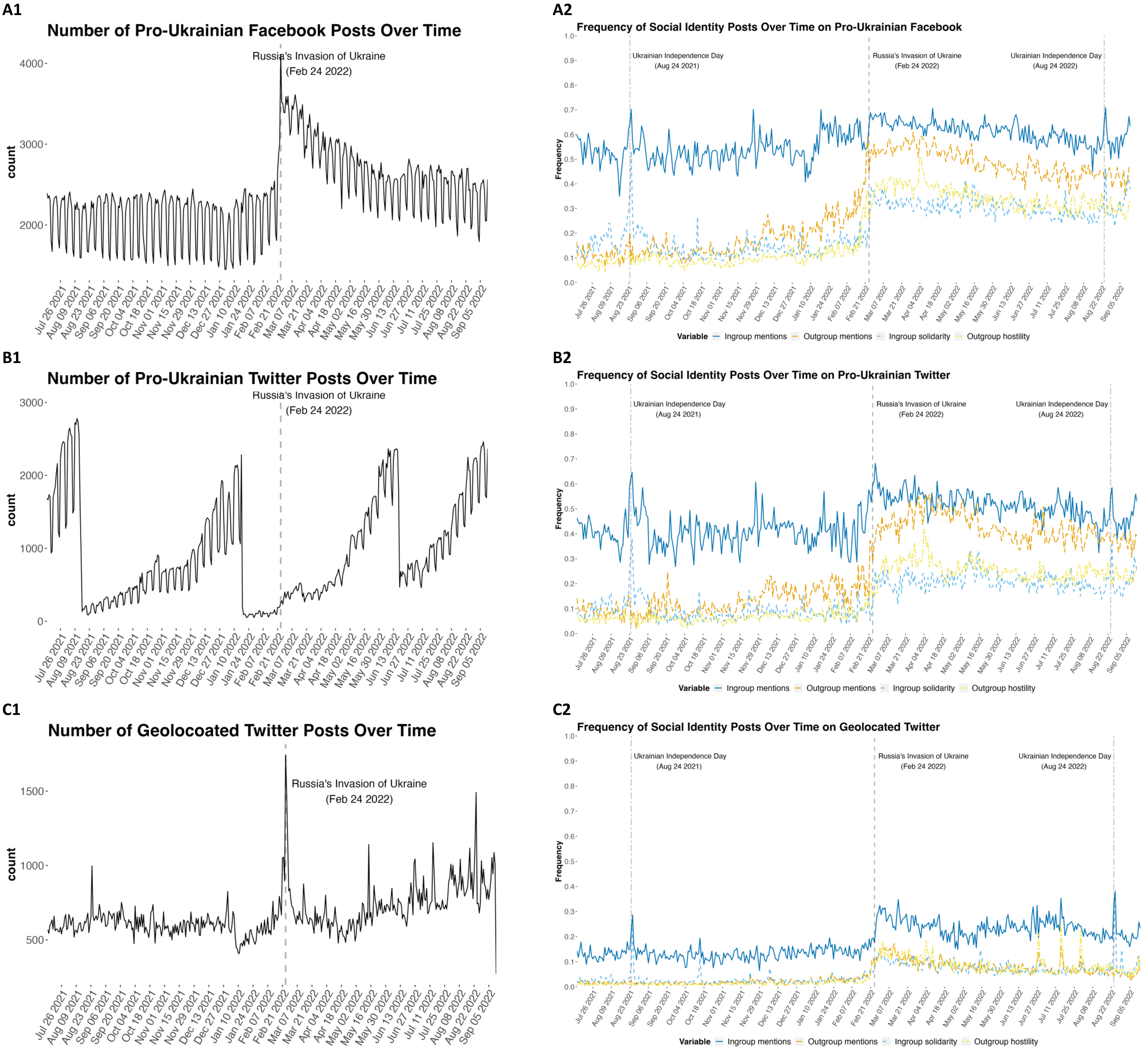

2.10. Supplementary Fig. 10. Distributions of posts and the proportion of posts classified as ingroup solidarity, outgroup hostility, and binary ingroup and outgroup mentions over time on (A) pro-Ukrainian Facebook news sources, (B) pro-Ukrainian Twitter news sources, (C) pro-Ukrainian Twitter geolocated.

Based on (A) N = 1, 011,171 Facebook posts, (B) N = 399,555 Twitter posts, (C) 289,592 Twitter posts.

Note for Supplementary Fig. 10 B1: We collected our news source data from Twitter using the non-academic API in four tranches (August 2021, January 2022, June 2022, and September 2022) by retrieving the 3200 most recent posts for each account using the R package "rtweet". This means that there is less data available just after data collection dates, as that data is the "oldest" at that point and falls outside of the rate limit of 3200 posts per page. For example, in January 2022, we collected data between August 2021 and January 2022. Because the API first retrieves the news tweets, we get 3200 tweets closest to January 2022, and if the page has posted more than that since August, some of the content does not get returned. Both the Twitter data and the Facebook data (which doesn't have this issue) show highly similar proportions of our variables of interest (the four different identity language categories), suggesting that the collected data is sufficiently representative (compare Supplementary Figures A2 and B2). It is, therefore, unlikely that the discrepancy in volume in Twitter data has a meaningful influence on our findings.

3. Supplementary Tables

3.1. Supplementary Table 1. Study 1: Predictors of engagement on Ukrainian social media before the invasion.

	Pro-Ukrainian Twitter	Pro-Russian Twitter	Pro-Ukrainian Facebook	Pro-Russian Facebook
(Intercept)	3.949 (t(62.635)=11.363, p<0.001, d=2.872, 95%CI=[3.116, 5.005])	16.163 (t(9.489)=2.384, p=0.040, d=1.548, 95%CI=[1.641, 159.210])	53.051 (t(76.671)=15.805, p<0.001, d=3.610, 95%CI=[32.421, 86.810])	62.942 (t(13.212)=14.621, p<0.001, d=8.045, 95%CI=[36.124, 109.671])
Ingroup mentions	1.156 (t(182031.995)=46.658, p<0.001, d=0.219, 95%CI=[1.149, 1.163])	1.041 (t(46676.896)=6.491, p<0.001, d=0.060, 95%CI=[1.028, 1.053])	1.113 (t(468248.478)=62.266, p<0.001, d=0.182, 95%CI=[1.109, 1.117])	1.071 (t(114542.360)=20.242, p<0.001, d=0.120, 95%CI=[1.064, 1.079])
Outgroup mentions	1.229 (t(182021.378)=44.930, p<0.001, d=0.211, 95%CI=[1.218, 1.240])	1.035 (t(46680.324)=4.142, p<0.001, d=0.038, 95%CI=[1.018, 1.052])	1.160 (t(466964.379)=63.890, p<0.001, d=0.187, 95%CI=[1.154, 1.165])	1.184 (t(114461.159)=41.373, p<0.001, d=0.245, 95%CI=[1.175, 1.194])
Moral emotional	1.046 (t(181985.703)=10.339, p<0.001, d=0.048, 95%CI=[1.037, 1.055])	1.030 (t(46680.266)=4.415, p<0.001, d=0.041, 95%CI=[1.017, 1.044])	1.062 (t(468202.389)=23.711, p<0.001, d=0.069, 95%CI=[1.057, 1.068])	1.062 (t(114532.693)=12.969, p<0.001, d=0.077, 95%CI=[1.052, 1.071])
Positive	1.037 (t(181991.405)=11.160, p<0.001, d=0.052, 95%CI=[1.030, 1.043])	1.049 (t(46676.888)=10.366, p<0.001, d=0.096, 95%CI=[1.040, 1.059])	1.017 (t(468221.426)=9.774, p<0.001, d=0.029, 95%CI=[1.014, 1.021])	1.024 (t(114532.670)=7.703, p<0.001, d=0.046, 95%CI=[1.018, 1.030])
Negative	1.030 (t(181994.881)=9.173, p<0.001, d=0.043, 95%CI=[1.024, 1.037])	1.002 (t(46675.871)=0.330, p=0.742, d=0.003, 95%CI=[0.992, 1.012])	1.021 (t(468239.989)=11.331, p<0.001, d=0.033, 95%CI=[1.017, 1.025])	1.046 (t(114531.800)=13.241, p<0.001, d=0.078, 95%CI=[1.039, 1.053])
Total tokens	1.096 (t(182043.609)=27.345, p<0.001, d=0.128, 95%CI=[1.089, 1.104])	1.037 (t(46677.915)=5.815, p<0.001, d=0.054, 95%CI=[1.024, 1.049])	0.930 (t(468244.268)=-19.499, p<0.001, d=0.057, 95%CI=[0.923, 0.937])	0.857 (t(114545.067)=-18.187, p<0.001, d=0.107, 95%CI=[0.843, 0.872])
Followers count	2.172 (t(7111.428)=15.783, p<0.001, d=0.374, 95%CI=[1.972, 2.391])	152.154 (t(315.482)=10.228, p<0.001, d=1.152, 95%CI=[58.087, 398.555])		
Is retweet	0.759 (t(182017.195)=-21.392, p<0.001, d=0.100, 95%CI=[0.740, 0.778])	0.328 (t(46675.763)=-25.946, p<0.001, d=0.240, 95%CI=[0.302, 0.357])		
Followers at posting			63.614 (t(4456.648)=32.441, p<0.001, d=0.972, 95%CI=[49.498, 81.755])	3.190 (t(141.311)=7.326, p<0.001, d=1.233, 95%CI=[2.339, 4.351])
Has media			1.439 (t(468212.745)=7.522, p<0.001, d=0.022, 95%CI=[1.309, 1.583])	0.957 (t(112653.153)=-0.663, p=0.507, d=0.004, 95%CI=[0.840, 1.090])
Has URL			0.660 (t(468209.663)=-8.676, p<0.001, d=0.025, 95%CI=[0.601, 0.725])	0.373 (t(113132.962)=-15.098, p<0.001, d=0.090, 95%CI=[0.328, 0.424])
N	182053	46705	468310	114557
N (accounts)	63	15	84	15
AIC	457521.985	122103.394	1573240.766	424120.376
BIC	457633.217	122199.662	1573373.449	424236.162
R2 (fixed)	0.258	0.533	0.689	0.330
R2 (total)	0.672	0.982	0.922	0.546

Mixed-effects linear regression. DV is log-transformed engagement. Estimates are exponentiated. P-values estimated using Satterthwaite d.f. Cohen’s d (d) estimated using effectsize::t_to_d(t,df_error). See the jtools package and Methods for more information.

3.2. Supplementary Table 2. Study 2: Predictors of engagement on Ukrainian social media after the invasion.

	Pro-Ukrainian Twitter	Pro-Ukrainian Facebook
(Intercept)	2.965 (t(55.159)=1.946, p=0.057, d=0.524, 95%CI=[0.992, 8.860])	41.790 (t(84.128)=15.238, p<0.001, d=3.323, 95%CI=[25.856, 67.543])
Ingroup mentions	1.113 (t(217179.930)=35.993, p<0.001, d=0.154, 95%CI=[1.107, 1.120])	1.043 (t(535713.511)=26.272, p<0.001, d=0.072, 95%CI=[1.040, 1.046])
Outgroup mentions	1.056 (t(217172.191)=17.364, p<0.001, d=0.075, 95%CI=[1.050, 1.063])	1.074 (t(535718.970)=41.712, p<0.001, d=0.114, 95%CI=[1.070, 1.077])
Moral emotional	1.053 (t(217167.057)=13.281, p<0.001, d=0.057, 95%CI=[1.045, 1.061])	1.040 (t(535705.994)=18.764, p<0.001, d=0.051, 95%CI=[1.036, 1.045])
Positive	1.069 (t(217166.170)=19.480, p<0.001, d=0.084, 95%CI=[1.062, 1.076])	1.034 (t(535707.445)=19.458, p<0.001, d=0.053, 95%CI=[1.031, 1.038])
Negative	1.001 (t(217166.422)=0.314, p=0.753, d=0.001, 95%CI=[0.995, 1.006])	1.001 (t(535713.376)=0.874, p=0.382, d=0.002, 95%CI=[0.998, 1.004])
Total tokens	1.059 (t(217183.913)=18.781, p<0.001, d=0.081, 95%CI=[1.053, 1.066])	0.892 (t(535746.805)=-36.489, p<0.001, d=0.100, 95%CI=[0.887, 0.898])
Followers count	0.019 (t(11054.598)=-23.450, p<0.001, d=0.446, 95%CI=[0.014, 0.026])	
Is retweet	0.935 (t(217177.506)=-4.360, p<0.001, d=0.019, 95%CI=[0.907, 0.964])	
Followers at posting		0.459 (t(522197.565)=-52.202, p<0.001, d=0.144, 95%CI=[0.446, 0.472])
Has media		0.950 (t(535713.027)=-2.575, p=0.010, d=0.007, 95%CI=[0.913, 0.988])
Has URL		0.423 (t(535712.837)=-43.721, p<0.001, d=0.119, 95%CI=[0.407, 0.440])
N	217245	535797
N (accounts)	63	84
AIC	607085.183	1840622.706
BIC	607198.359	1840757.004
R2 (fixed)	0.458	0.105
R2 (total)	0.975	0.761

Mixed-effects linear regression. DV is log-transformed engagement. Estimates are exponentiated. P-values estimated using Satterthwaite d.f. Cohen's d (d) estimated using effectsize::t_to_d(t,df_error). See the jtools package and Methods for more information.

3.3. Supplementary Table 3. Study 2: Predictors of engagement on Ukrainian social media with ingroup solidarity and outgroup hostility.

	Pro-Ukrainian Twitter Before	Pro-Ukrainian Twitter After	Pro-Ukrainian Facebook Before	Pro-Ukrainian Facebook After
(Intercept)	3.422 (t(62.767)=10.391, p<0.001, d=2.623, 95%CI=[2.713, 4.315])	2.257 (t(55.692)=1.394, p=0.169, d=0.374, 95%CI=[0.719, 7.091])	42.792 (t(76.330)=14.730, p<0.001, d=3.372, 95%CI=[25.960, 70.538])	33.622 (t(84.259)=14.659, p<0.001, d=3.194, 95%CI=[21.014, 53.794])
Ingroup mentions (binary)	1.162 (t(180315.248)=34.722, p<0.001, d=0.164, 95%CI=[1.152, 1.172])	1.073 (t(214820.451)=16.172, p<0.001, d=0.070, 95%CI=[1.064, 1.083])	1.184 (t(465918.453)=40.891, p<0.001, d=0.120, 95%CI=[1.174, 1.194])	0.966 (t(533142.181)=-8.684, p<0.001, d=0.024, 95%CI=[0.958, 0.974])
Outgroup mentions (binary)	1.377 (t(180338.540)=46.864, p<0.001, d=0.221, 95%CI=[1.358, 1.395])	1.160 (t(214808.090)=32.402, p<0.001, d=0.140, 95%CI=[1.150, 1.171])	1.396 (t(465007.134)=55.533, p<0.001, d=0.163, 95%CI=[1.380, 1.413])	1.198 (t(533144.519)=44.846, p<0.001, d=0.123, 95%CI=[1.189, 1.208])
Ingroup solidarity	1.517 (t(180299.583)=55.804, p<0.001, d=0.263, 95%CI=[1.495, 1.539])	1.684 (t(214803.124)=96.314, p<0.001, d=0.416, 95%CI=[1.666, 1.702])	1.612 (t(465909.097)=82.514, p<0.001, d=0.242, 95%CI=[1.594, 1.630])	1.925 (t(533141.026)=151.945, p<0.001, d=0.416, 95%CI=[1.909, 1.941])
Outgroup hostility	1.117 (t(180297.355)=12.017, p<0.001, d=0.057, 95%CI=[1.097, 1.137])	0.994 (t(214803.461)=-1.114, p=0.265, d=0.005, 95%CI=[0.984, 1.005])	1.100 (t(465871.262)=12.777, p<0.001, d=0.037, 95%CI=[1.084, 1.116])	1.014 (t(533139.509)=3.319, p=0.001, d=0.009, 95%CI=[1.006, 1.023])
Moral emotional	1.035 (t(180298.880)=7.961, p<0.001, d=0.037, 95%CI=[1.026, 1.044])	1.015 (t(214803.186)=3.909, p<0.001, d=0.017, 95%CI=[1.007, 1.023])	1.048 (t(465872.219)=18.309, p<0.001, d=0.054, 95%CI=[1.042, 1.053])	1.014 (t(533137.966)=6.809, p<0.001, d=0.019, 95%CI=[1.010, 1.018])
Positive	1.007 (t(180305.394)=2.163, p=0.031, d=0.010, 95%CI=[1.001, 1.013])	1.016 (t(214802.736)=4.657, p<0.001, d=0.020, 95%CI=[1.009, 1.023])	0.989 (t(465897.019)=-6.370, p<0.001, d=0.019, 95%CI=[0.985, 0.992])	0.989 (t(533139.669)=-6.528, p<0.001, d=0.018, 95%CI=[0.985, 0.992])
Negative	1.025 (t(180308.011)=7.594, p<0.001, d=0.036, 95%CI=[1.018, 1.031])	0.999 (t(214803.130)=-0.537, p=0.591, d=0.002, 95%CI=[0.993, 1.004])	1.020 (t(465899.753)=10.914, p<0.001, d=0.032, 95%CI=[1.017, 1.024])	1.000 (t(533146.001)=-0.122, p=0.903, d=0.000, 95%CI=[0.997, 1.003])
Total tokens	1.089 (t(180356.350)=25.779, p<0.001, d=0.121, 95%CI=[1.082, 1.096])	1.059 (t(214820.482)=19.502, p<0.001, d=0.084, 95%CI=[1.053, 1.066])	0.999 (t(465893.892)=-0.290, p=0.772, d=0.001, 95%CI=[0.992, 1.006])	0.957 (t(533179.474)=-15.169, p<0.001, d=0.042, 95%CI=[0.952, 0.963])
Followers count	2.103 (t(6844.478)=15.302, p<0.001, d=0.370, 95%CI=[1.912, 2.314])	0.014 (t(13383.753)=-25.127, p<0.001, d=0.434, 95%CI=[0.010, 0.020])		
Followers at posting			67.335 (t(4688.811)=32.800, p<0.001, d=0.958, 95%CI=[52.360, 86.595])	0.465 (t(519473.271)=-52.049, p<0.001, d=0.144, 95%CI=[0.452, 0.479])
Has media			1.396 (t(465881.941)=6.930, p<0.001, d=0.020, 95%CI=[1.270, 1.534])	0.851 (t(533145.041)=-8.195, p<0.001, d=0.022, 95%CI=[0.819, 0.885])
Has URL			0.669 (t(465879.501)=-8.449, p<0.001, d=0.025, 95%CI=[0.609, 0.734])	0.416 (t(533144.821)=-45.385, p<0.001, d=0.124, 95%CI=[0.401, 0.433])
N	180367	214882	465982	533231
N (accounts)	63	63	84	84
AIC	449293.112	591046.739	1556941.633	1809517.419
BIC	449414.345	591170.074	1557096.360	1809674.033
R2 (fixed)	0.260	0.468	0.692	0.111
R2 (total)	0.669	0.978	0.926	0.763

Mixed-effects linear regression. DV is log-transformed engagement. Estimates are exponentiated. P-values estimated using Satterthwaite d.f. Cohen's d (d) estimated using effectsize::t_to_d(t,df_error). See the jtools package and Methods for more information. Moral emotional, positive and negative variables represent word counts and their coefficients can be compared with each other but not with the binary categories.

3.4. Supplementary Table 4. Study 3: Predictors of engagement on geolocated Ukrainian Twitter before and after the invasion.

	Pro-Ukrainian Geolocated Twitter Before	Pro-Ukrainian Geolocated Twitter After
(Intercept)	2.154 (t(7346.665)=35.376, p<0.001, d=0.825, 95%CI=[2.065, 2.248])	2.210 (t(9699.179)=48.811, p<0.001, d=0.991, 95%CI=[2.141, 2.282])
Ingroup mentions (binary)	1.067 (t(135294.944)=9.231, p<0.001, d=0.050, 95%CI=[1.052, 1.082])	1.071 (t(147015.169)=10.999, p<0.001, d=0.057, 95%CI=[1.058, 1.085])
Outgroup mentions (binary)	1.080 (t(133583.109)=5.135, p<0.001, d=0.028, 95%CI=[1.048, 1.112])	1.044 (t(146191.418)=4.873, p<0.001, d=0.025, 95%CI=[1.026, 1.063])
Ingroup solidarity	1.289 (t(134818.891)=18.880, p<0.001, d=0.103, 95%CI=[1.255, 1.323])	1.144 (t(147612.576)=14.849, p<0.001, d=0.077, 95%CI=[1.124, 1.164])
Outgroup hostility	1.146 (t(132982.352)=7.974, p<0.001, d=0.044, 95%CI=[1.108, 1.185])	1.069 (t(145861.552)=8.009, p<0.001, d=0.042, 95%CI=[1.051, 1.086])
Positive	1.015 (t(133605.662)=5.944, p<0.001, d=0.033, 95%CI=[1.010, 1.021])	1.004 (t(146345.954)=1.529, p=0.126, d=0.008, 95%CI=[0.999, 1.010])
Negative	1.000 (t(132822.741)=-0.050, p=0.960, d=0.000, 95%CI=[0.993, 1.007])	0.986 (t(145580.091)=-4.274, p<0.001, d=0.022, 95%CI=[0.980, 0.993])
Moral emotional	1.016 (t(133128.576)=3.308, p=0.001, d=0.018, 95%CI=[1.006, 1.025])	1.018 (t(146112.935)=4.073, p<0.001, d=0.021, 95%CI=[1.009, 1.027])
Total tokens	1.068 (t(133925.893)=25.132, p<0.001, d=0.137, 95%CI=[1.063, 1.074])	1.085 (t(146534.951)=28.390, p<0.001, d=0.148, 95%CI=[1.079, 1.091])
Follower count	1.177 (t(6155.908)=9.385, p<0.001, d=0.239, 95%CI=[1.137, 1.218])	1.030 (t(14854.668)=7.457, p<0.001, d=0.122, 95%CI=[1.022, 1.038])
User is not verified	0.893 (t(6884.451)=-4.335, p<0.001, d=0.105, 95%CI=[0.848, 0.940])	1.003 (t(8019.455)=0.134, p=0.893, d=0.003, 95%CI=[0.955, 1.054])
User is verified	2.990 (t(5962.132)=9.963, p<0.001, d=0.258, 95%CI=[2.410, 3.709])	7.238 (t(7126.605)=16.363, p<0.001, d=0.388, 95%CI=[5.710, 9.175])
Has URL	1.137 (t(136090.096)=23.777, p<0.001, d=0.129, 95%CI=[1.125, 1.150])	1.003 (t(147558.743)=0.465, p=0.642, d=0.002, 95%CI=[0.992, 1.014])
N	136950	149230
N (author_id)	6446	7596
AIC	308426.906	371370.649
BIC	308574.317	371519.348
R2 (fixed)	0.040	0.050
R2 (total)	0.615	0.594

Mixed-effects linear regression. DV is log-transformed engagement. Estimates are exponentiated. P-values estimated using Satterthwaite d.f. Cohen's d (d) estimated using effectsize::t_to_d(t,df_error). See the jtools package and Methods for more information. Moral emotional, positive and negative variables represent word counts and their coefficients can be compared with each other but not with the binary categories. The RoBERTa classifier used in Study 3 to label posts as pro-Ukrainian or pro-Russian was trained and evaluated exclusively on the data from after the invasion and thus does not necessarily generalize to the prior period. Therefore, we cannot make any claims concerning the predictive power of these four variables for Twitter engagement for the period before the invasion. However, we provide this data for completeness (see SI Tables S3 and S7). "user is not verified" (verifiedFalse) and "user is verified" (verifiedTrue) variables together indicate one of three possibilities: 1) Twitter user has a verification tag (verifiedTrue); 2) Twitter user has no verification tag (verifiedFalse); or 3) user does not exist or has been removed (NULL).

3.5. Supplementary Table 5. Descriptive Statistics for pro-Ukrainian news sources on Facebook before and after the invasion.

	Pro-Ukrainian Facebook Before					Pro-Ukrainian Facebook After				
	N	Mean	St. Dev.	Min	Max	N	Mean	St. Dev.	Min	Max
ingroup mentions	468,729	1.057	1.38	0	36	533,370	1.196	1.422	0	43
outgroup mentions	468,729	0.322	0.864	0	36	533,370	0.869	1.226	0	50
ingroup mentions (binary)	468,729	0.543	0.498	0	1	533,370	0.614	0.487	0	1
outgroup mentions (binary)	468,729	0.172	0.377	0	1	533,370	0.488	0.5	0	1
ingroup solidarity	468,729	0.146	0.353	0	1	533,370	0.306	0.461	0	1
outgroup hostility	468,729	0.102	0.303	0	1	533,370	0.347	0.476	0	1
positive	468,729	0.809	1.299	0	51	533,370	0.821	1.432	0	59
negative	468,729	0.815	1.264	0	49	533,370	1.334	1.739	0	63
moral-emotional	468,729	0.454	0.876	0	21	533,370	0.797	1.204	0	42
total tokens	468,729	27.543	21.358	1	864	533,370	31.198	31.969	1	890
followers at posting	465,982	407,511.90	547,835.00	682	2,214,479	533,231	512,734.20	623,022.20	704	2,379,253
has URL	468,729	0.892	0.31	0	1	533,370	0.851	0.356	0	1
has media	468,729	0.106	0.308	0	1	533,370	0.139	0.346	0	1
engagement (log-transformed)	468,729	2.682	2.084	0	13.451	533,370	3.882	2.235	0	13.351

3.6. Supplementary Table 6. Descriptive Statistics for pro-Ukrainian news sources on Twitter before and after the invasion.

	Pro-Ukrainian Twitter Before					Pro-Ukrainian Twitter After				
	N	Mean	St. Dev.	Min	Max	N	Mean	St. Dev.	Min	Max
ingroup	180,367	0.518	0.735	0	8	214,882	0.638	0.764	0	7
outgroup	180,367	0.153	0.451	0	6	214,882	0.516	0.745	0	7
ingroup mentions (binary)	180,367	0.41	0.492	0	1	214,882	0.5	0.5	0	1
outgroup mentions (binary)	180,367	0.124	0.329	0	1	214,882	0.406	0.491	0	1
ingroup solidarity	180,367	0.086	0.28	0	1	214,882	0.209	0.406	0	1
outgroup hostility	180,367	0.063	0.243	0	1	214,882	0.246	0.431	0	1
positive	180,367	0.356	0.667	0	12	214,882	0.346	0.654	0	9
negative	180,367	0.399	0.703	0	8	214,882	0.721	0.959	0	10
moral-emotional	180,367	0.228	0.509	0	8	214,882	0.398	0.661	0	9
total tokens	180,367	13.442	6.965	1	53	214,882	15.295	7.958	1	55
follower count	180,367	151,626.00	253,531.90	409	888,352	214,882	194,687.10	281,169.60	105	1,014,571
has URL	180,367	1	0	1	1	214,882	1	0	1	1
has media	180,367	1	0	1	1	214,882	1	0	1	1
engagement (log-transformed)	180,367	1.47	1.345	0	7.931	214,882	2.478	1.734	0	10.9

3.7. Supplementary Table 7. Descriptive Statistics for pro-Russian news sources on Facebook and Twitter (before the invasion).

	Pro-Russian Facebook					Pro-Russian Twitter				
	N	Mean	St. Dev.	Min	Max	N	Mean	St. Dev.	Min	Max
ingroup	114,028	0.782	1.626	0	41	46,412	0.411	0.689	0	7
outgroup	114,028	0.551	1.311	0	36	46,412	0.344	0.734	0	6
positive	114,028	1.507	2.238	0	52	46,412	0.757	1.005	0	8
negative	114,028	1.103	1.801	0	36	46,412	0.587	0.878	0	10
moral-emotional	114,028	0.713	1.291	0	29	46,412	0.376	0.668	0	6
total tokens	114,028	43.22	44.362	1	784	46,412	22.482	10.386	1	52
followers at posting / follower count	114,028	929,384.50	715,553.70	65,096	2,276,005	46,412	824,808.00	990,881.30	246	3,008,358
has URL	114,028	0.631	0.482	0	1	46,412	1	0	1	1
has media	114,028	0.364	0.481	0	1	46,412	1	0	1	1
engagement (log-transformed)	114,028	3.427	1.866	0	12.437	46,412	2.176	1.395	0	8.642

3.8. Supplementary Table 8. Descriptive Statistics for pro-Ukrainian geolocated Twitter before and after the invasion.

The RoBERTa classifier used in Study 3 to label posts as pro-Ukrainian or pro-Russian was trained and evaluated exclusively on the data from after the invasion and thus does not necessarily generalize to the prior period. Therefore, we cannot make any claims concerning the predictive power of these four variables for Twitter engagement for the period before the invasion. However, we provide this data for completeness (see SI Tables S3 and S7).

	Pro-Ukrainian Geolocated Twitter Before					**Pro-Ukrainian Geolocated Twitter After**				
	N	Mean	St. Dev.	Min	Max	N	Mean	St. Dev.	Min	Max
ingroup mentions (binary)	136,950	0.134	0.341	0	1	149,230	0.238	0.426	0	1
outgroup mentions (binary)	136,950	0.022	0.145	0	1	149,230	0.08	0.271	0	1
ingroup solidarity	136,950	0.027	0.163	0	1	149,230	0.082	0.274	0	1
outgroup hostility	136,950	0.015	0.122	0	1	149,230	0.087	0.282	0	1
positive	136,950	0.54	0.891	0	14	149,230	0.492	0.871	0	12
negative	136,950	0.271	0.627	0	10	149,230	0.42	0.811	0	20
moral-emotional	136,950	0.175	0.467	0	8	149,230	0.25	0.583	0	13
total tokens	136,950	14.551	10.704	1	60	149,230	14.827	11.176	1	90
follower count	94,795	2,993.91	15,728.54	0	1,331,921	77,251	4,698.40	41,679.38	0	9,329,320
user is verified (binary)	94,795	0.027	0.161	0	1	77,251	0.03	0.17	0	1
has URL	136,950	0.555	0.497	0	1	149,230	0.628	0.483	0	1
engagement (log-transformed)	136,950	1.289	1.323	0	8.337	149,230	1.34	1.481	0	10.204

3.9. Supplementary Table 9. Correlation matrix for pro-Ukrainian news sources on Facebook.

	ingroup	outgroup	ingroup mentions (binary)	outgroup mentions (binary)	ingroup solidarity	outgroup hostility	ingroup solidarity (binary)	outgroup hostility (binary)	positive	negative	moral-emotional
ingroup mentions	1										
outgroup mentions	0.13	1									
ingroup mentions (binary)	0.68	0.04	1								
outgroup mentions (binary)	0.04	0.77	0.03	1							
ingroup solidarity	0.26	0.03	0.25	0.05	1						
outgroup hostility	-0.01	0.42	-0.01	0.47	0.08	1					
ingroup solidarity (binary)	0.14	0.04	0.1	0.03	0.33	0.04	1				
outgroup hostility (binary)	0.02	0.1	0.01	0.15	0.11	0.41	0.08	1			
positive	0.27	0.1	0.08	0.01	0.25	-0.02	0.21	0.02	1		
negative	0.22	0.31	0.11	0.22	0.11	0.28	0.13	0.26	0.21	1	
moral-emotional	0.21	0.25	0.09	0.18	0.18	0.19	0.15	0.2	0.33	0.59	1

3.10. Supplementary Table 10. Correlation matrix for pro-Ukrainian news sources on Twitter.

	ingroup	outgroup	ingroup mentions (binary)	outgroup mentions (binary)	ingroup solidarity	outgroup hostility	ingroup solidarity (binary)	outgroup hostility (binary)	positive	negative	moral-emotional
ingroup mentions	1										
outgroup mentions	0.11	1									
ingroup mentions (binary)	0.84	0.06	1								
outgroup mentions (binary)	0.05	0.87	0.03	1							
ingroup solidarity	0.26	0.03	0.25	0.03	1						
outgroup hostility	0.04	0.41	0.02	0.39	0.07	1					
ingroup solidarity (binary)	0.07	0.01	0.06	0.01	0.25	0.01	1				
outgroup hostility (binary)	0	0.06	0	0.07	0.07	0.35	0.02	1			
positive	0.07	0.02	0.04	-0.01	0.18	-0.02	0.13	-0.03	1		
negative	0.14	0.29	0.09	0.22	0.1	0.27	0.03	0.21	0.04	1	
moral-emotional	0.06	0.14	0.04	0.14	0.14	0.15	0.05	0.15	0.16	0.5	1

3.11. Supplementary Table 11. Correlation matrix for geolocated pro-Ukrainian Twitter.

	ingroup	outgroup	ingroup mentions (binary)	outgroup mentions (binary)	ingroup solidarity	outgroup hostility	positive	negative	moral-emotional
ingroup mentions	1								
outgroup mentions	0.15	1							
ingroup mentions (binary)	0.88	0.15	1						
outgroup mentions (binary)	0.16	0.91	0.15	1					
ingroup solidarity	0.33	0.07	0.33	0.07	1				
outgroup hostility	0.05	0.32	0.05	0.33	0.05	1			
positive	0.06	0.03	0.04	0.02	0.2	0	1		
negative	0.08	0.16	0.07	0.16	0.07	0.18	0.13	1	
moral-emotional	0.1	0.15	0.09	0.14	0.14	0.13	0.29	0.43	1

3.12. Supplementary Table 12. Descriptive statistics of Facebook reactions for pro-Ukrainian news sources before the invasion.

	Shares	Likes	Comments	Love	Wow	Haha	Sad	Angry	Mean	SD
Shares	1								15.545	328.554
Likes	0.57	1							104.397	1,301.664
Comments	0.41	0.46	1						23.051	153.165
Love	0.41	0.9	0.33	1					6.904	121.144
Wow	0.28	0.2	0.28	0.11	1				3.596	36.751
Haha	0.18	0.13	0.43	0.06	0.19	1			10.192	74.170
Sad	0.26	0.15	0.22	0.03	0.17	0.01	1		16.043	287.353
Angry	0.27	0.12	0.62	0.05	0.19	0.21	0.14	1	7.354	79.499

3.13. Supplementary Table 13. Descriptive statistics of Facebook reactions for pro-Ukrainian news sources after the invasion.

	Shares	Likes	Comments	Love	Wow	Haha	Sad	Angry	Mean	SD
Shares	1								45.110	633.529
Likes	0.64	1							363.775	2,608.479
Comments	0.51	0.4	1						35.378	333.894
Love	0.46	0.88	0.28	1					29.237	273.113
Wow	0.21	0.18	0.32	0.11	1				2.913	32.579
Haha	0.15	0.15	0.44	0.09	0.24	1			12.738	165.202
Sad	0.34	0.23	0.29	0.02	0.07	0	1		67.529	1,417.101
Angry	0.36	0.09	0.71	0.02	0.22	0.24	0.11	1	17.802	263.519

3.14. Supplementary Table 14. Descriptive statistics of Facebook reactions for pro-Russian news sources before the invasion.

	Shares	Likes	Comments	Love	Wow	Haha	Sad	Angry	Mean	SD
Shares	1								16.587	301.009
Likes	0.68	1							134.786	1,749.616
Comments	0.39	0.39	1						41.678	216.638
Love	0.6	0.87	0.35	1					10.819	153.079
Wow	0.37	0.25	0.27	0.12	1				5.115	39.196
Haha	0.34	0.26	0.35	0.13	0.37	1			14.577	89.644
Sad	0.25	0.31	0.28	0.12	0.22	0.02	1		14.125	222.138
Angry	0.19	0.12	0.71	0.08	0.17	0.13	0.15	1	7.680	98.736

3.15. Supplementary Table 15. Classifier validation results (Facebook and Twitter news sources). Results are based on a dataset of 400 posts stratified by class (different for ingroup solidarity and outgroup hostility).

	F1 Macro (average of F1 per class)	Balanced Accuracy (average of recall per class)	Accuracy (imbalanced; proportion of correctly classified)
**Ingroup Mentions**			
Dictionary	0.759	0.788	0.759
Untrained Ukrainian and Russian speaker	0.832	0.82	0.847
**Outgroup Mentions**			
Dictionary	0.872	0.848	0.9
Untrained Ukrainian and Russian speaker	0.893	0.92	0.905
**Ingroup Solidarity**			
Dictionary	0.576	0.569	0.782
Untrained Ukrainian and Russian speaker	0.665	0.634	0.842
BERT-NLI	0.79	0.772	0.868
**Outgroup Hostility**			
Dictionary	0.66	0.654	0.795
Untrained Ukrainian and Russian speaker	0.731	0.689	0.868
BERT-NLI	0.805	0.819	0.873

3.16. Supplementary Table 16. Classifier validation results (geolocated Twitter). Results for everything but pro-Ukrainian classifier are based on the data classified as pro-Ukrainian by the model (309 posts).

	F1 Macro (average of F1 per class)	Balanced Accuracy (average of recall per class)	Accuracy (imbalanced; proportion of correctly classified)
Pro-Ukrainian RoBERTa	0.812	0.798	0.86
Ingroup Mentions (Dictionary)	0.833	0.802	0.877
Outgroup Mentions (Dictionary)	0.83	0.786	0.958
**Ingroup Solidarity**			
Dictionary	0.602	0.676	0.854
BERT-NLI (finetuned)	0.748	0.748	0.942
**Outgroup Hostility**			
Dictionary	0.692	0.683	0.929
BERT-NLI (finetuned)	0.821	0.836	0.955

3.17. Supplementary Table 17. Study 1: Predictors of Twitter reactions (RT and Favorite) on Ukrainian social media before and after the invasion.

	RT Pro-Ukrainian Twitter Before	RT Pro-Russian Twitter Before	Favorite Pro-Ukrainian Twitter Before	Favorite Pro-Russian Twitter Before	RT Pro-Ukrainian Twitter After	Favorite Pro-Ukrainian Twitter After
(Intercept)	2.141 (t(62.072)=9.453, p<0.001, d=2.400, 95%CI=[1.829, 2.508])	6.640 (t(11.179)=1.373, p=0.197, d=0.821, 95%CI=[0.445, 99.125])	3.211 (t(62.564)=10.495, p<0.001, d=2.654, 95%CI=[2.582, 3.992])	12.111 (t(8.660)=2.366, p=0.043, d=1.608, 95%CI=[1.534, 95.609])	0.710 (t(58.992)=-0.464, p=0.644, d=0.121, 95%CI=[0.167, 3.020])	2.524 (t(51.655)=2.021, p=0.048, d=0.562, 95%CI=[1.028, 6.196])
Ingroup mentions	1.102 (t(182043.648)=35.995, p<0.001, d=0.169, 95%CI=[1.096, 1.107])	1.015 (t(46679.311)=2.673, p=0.008, d=0.025, 95%CI=[1.004, 1.026])	1.150 (t(182034.650)=46.629, p<0.001, d=0.219, 95%CI=[1.143, 1.157])	1.043 (t(46675.326)=6.874, p<0.001, d=0.064, 95%CI=[1.031, 1.056])	1.048 (t(217176.396)=18.705, p<0.001, d=0.080, 95%CI=[1.043, 1.053])	1.127 (t(217182.058)=39.400, p<0.001, d=0.169, 95%CI=[1.121, 1.134])
Outgroup mentions	1.183 (t(182034.333)=42.466, p<0.001, d=0.199, 95%CI=[1.174, 1.192])	1.025 (t(46681.162)=3.388, p=0.001, d=0.031, 95%CI=[1.011, 1.040])	1.186 (t(182023.699)=38.598, p<0.001, d=0.181, 95%CI=[1.175, 1.196])	1.027 (t(46679.719)=3.145, p=0.002, d=0.029, 95%CI=[1.010, 1.044])	1.067 (t(217173.153)=24.826, p<0.001, d=0.107, 95%CI=[1.062, 1.073])	1.037 (t(217170.516)=11.265, p<0.001, d=0.048, 95%CI=[1.030, 1.043])
Moral emotional	1.045 (t(181987.413)=11.696, p<0.001, d=0.055, 95%CI=[1.037, 1.053])	1.025 (t(46681.257)=4.032, p<0.001, d=0.037, 95%CI=[1.013, 1.037])	1.025 (t(181985.951)=5.859, p<0.001, d=0.027, 95%CI=[1.016, 1.033])	1.028 (t(46679.489)=4.038, p<0.001, d=0.037, 95%CI=[1.014, 1.042])	1.039 (t(217171.076)=11.858, p<0.001, d=0.051, 95%CI=[1.033, 1.046])	1.049 (t(217162.630)=12.167, p<0.001, d=0.052, 95%CI=[1.041, 1.058])
Positive	0.988 (t(181994.373)=-4.256, p<0.001, d=0.020, 95%CI=[0.983, 0.994])	1.003 (t(46679.269)=0.718, p=0.473, d=0.007, 95%CI=[0.995, 1.011])	1.048 (t(181991.899)=15.123, p<0.001, d=0.071, 95%CI=[1.042, 1.054])	1.067 (t(46675.365)=13.998, p<0.001, d=0.130, 95%CI=[1.057, 1.077])	1.002 (t(217170.723)=0.765, p=0.444, d=0.003, 95%CI=[0.997, 1.008])	1.077 (t(217161.250)=21.359, p<0.001, d=0.092, 95%CI=[1.070, 1.085])
Negative	1.053 (t(182002.029)=18.586, p<0.001, d=0.087, 95%CI=[1.048, 1.059])	1.033 (t(46678.741)=7.068, p<0.001, d=0.065, 95%CI=[1.024, 1.042])	1.010 (t(181995.926)=3.069, p=0.002, d=0.014, 95%CI=[1.003, 1.016])	0.984 (t(46674.039)=-3.177, p=0.001, d=0.029, 95%CI=[0.974, 0.994])	1.009 (t(217170.820)=3.971, p<0.001, d=0.017, 95%CI=[1.005, 1.014])	0.993 (t(217161.651)=-2.394, p=0.017, d=0.010, 95%CI=[0.987, 0.999])
Total tokens	1.105 (t(182004.331)=34.359, p<0.001, d=0.161, 95%CI=[1.099, 1.111])	1.051 (t(46679.767)=8.961, p<0.001, d=0.083, 95%CI=[1.039, 1.062])	1.047 (t(182041.975)=14.232, p<0.001, d=0.067, 95%CI=[1.041, 1.054])	1.026 (t(46676.750)=4.122, p<0.001, d=0.038, 95%CI=[1.014, 1.039])	1.105 (t(217177.930)=39.288, p<0.001, d=0.169, 95%CI=[1.100, 1.111])	1.007 (t(217188.433)=2.270, p=0.023, d=0.010, 95%CI=[1.001, 1.013])
Followers count	1.735 (t(2823.091)=13.389, p<0.001, d=0.504, 95%CI=[1.601, 1.881])	312.684 (t(991.048)=12.658, p<0.001, d=0.804, 95%CI=[128.459, 761.112])	2.257 (t(6035.038)=17.288, p<0.001, d=0.445, 95%CI=[2.058, 2.475])	91.230 (t(203.410)=9.330, p<0.001, d=1.308, 95%CI=[35.347, 235.464])	0.002 (t(55597.798)=-42.662, p<0.001, d=0.362, 95%CI=[0.002, 0.003])	0.045 (t(4716.806)=-18.256, p<0.001, d=0.532, 95%CI=[0.032, 0.063])
Is retweet	1.670 (t(182032.647)=45.991, p<0.001, d=0.216, 95%CI=[1.634, 1.707])	1.336 (t(46678.677)=7.599, p<0.001, d=0.070, 95%CI=[1.240, 1.440])	0.222 (t(182019.756)=-121.331, p<0.001, d=0.569, 95%CI=[0.216, 0.227])	0.051 (t(46673.907)=-69.129, p<0.001, d=0.640, 95%CI=[0.047, 0.055])	2.451 (t(217175.121)=69.653, p<0.001, d=0.299, 95%CI=[2.390, 2.513])	0.107 (t(217179.314)=-141.907, p<0.001, d=0.609, 95%CI=[0.104, 0.110])
N	182053	46705	182053	46705	217245	217245
N (user_id)	63	15	63	15	63	63
AIC	404201.087	111064.499	443599.990	122537.391	528181.046	616085.195
BIC	404312.319	111160.767	443711.222	122633.658	528294.223	616198.372
R2 (fixed)	0.265	0.520	0.300	0.530	0.540	0.442
R2 (total)	0.578	0.990	0.674	0.978	0.991	0.961

Mixed-effects linear regression. DV is log-transformed engagement. Estimates are exponentiated. P-values estimated using Satterthwaite d.f. Cohen's d (d) estimated using effectsize::t_to_d(t,df_error). See the jtools package and Methods for more information.

3.18. Supplementary Table 18. Study 1: Predictors of Facebook reactions on Ukrainian social media before the invasion (pro-Ukrainian).

	Share Pro-Ukrainian Facebook Before	Like Pro-Ukrainian Facebook Before	Comment Pro-Ukrainian Facebook Before	Love Pro-Ukrainian Facebook Before	Wow Pro-Ukrainian Facebook Before	Haha Pro-Ukrainian Facebook Before	Sad Pro-Ukrainian Facebook Before	Angry Pro-Ukrainian Facebook Before
(Intercept)	2.921 (t(78.674)=12.674, p<0.001, d=2.858, 95%CI=[2.475, 3.448])	36.951 (t(76.392)=9.388, p<0.001, d=2.148, 95%CI=[17.393, 78.503])	5.170 (t(64.578)=14.543, p<0.001, d=3.619, 95%CI=[4.143, 6.452])	1.863 (t(62.206)=11.308, p<0.001, d=2.867, 95%CI=[1.672, 2.075])	1.450 (t(44.125)=4.882, p<0.001, d=1.470, 95%CI=[1.249, 1.683])	2.877 (t(51.278)=13.935, p<0.001, d=3.892, 95%CI=[2.480, 3.338])	1.083 (t(56.155)=0.547, p=0.587, d=0.146, 95%CI=[0.814, 1.442])	2.048 (t(79.855)=16.154, p<0.001, d=3.615, 95%CI=[1.877, 2.234])
Ingroup mentions	1.024 (t(468301.692)=18.079, p<0.001, d=0.053, 95%CI=[1.021, 1.026])	1.100 (t(468231.592)=56.261, p<0.001, d=0.164, 95%CI=[1.096, 1.104])	1.114 (t(468297.812)=69.550, p<0.001, d=0.203, 95%CI=[1.111, 1.117])	1.061 (t(468278.814)=51.369, p<0.001, d=0.150, 95%CI=[1.059, 1.064])	0.986 (t(468296.335)=-13.278, p<0.001, d=0.039, 95%CI=[0.984, 0.988])	1.090 (t(468292.019)=59.264, p<0.001, d=0.173, 95%CI=[1.087, 1.093])	0.982 (t(468260.445)=-13.969, p<0.001, d=0.041, 95%CI=[0.979, 0.984])	1.034 (t(468103.342)=24.884, p<0.001, d=0.073, 95%CI=[1.031, 1.036])
Outgroup mentions	1.028 (t(459748.023)=15.475, p<0.001, d=0.046, 95%CI=[1.024, 1.031])	1.138 (t(468178.182)=56.492, p<0.001, d=0.165, 95%CI=[1.133, 1.143])	1.113 (t(459083.879)=51.023, p<0.001, d=0.151, 95%CI=[1.108, 1.117])	1.035 (t(457278.717)=21.915, p<0.001, d=0.065, 95%CI=[1.032, 1.038])	0.993 (t(454817.305)=-4.944, p<0.001, d=0.015, 95%CI=[0.990, 0.996])	1.162 (t(454458.687)=76.868, p<0.001, d=0.228, 95%CI=[1.157, 1.166])	0.919 (t(464129.605)=-47.783, p<0.001, d=0.140, 95%CI=[0.916, 0.922])	1.050 (t(462875.897)=27.209, p<0.001, d=0.080, 95%CI=[1.046, 1.054])
Moral emotional	1.034 (t(468218.789)=17.103, p<0.001, d=0.050, 95%CI=[1.030, 1.038])	1.053 (t(468211.300)=20.475, p<0.001, d=0.060, 95%CI=[1.048, 1.058])	1.027 (t(468198.345)=11.414, p<0.001, d=0.033, 95%CI=[1.022, 1.031])	1.023 (t(468207.912)=13.240, p<0.001, d=0.039, 95%CI=[1.020, 1.027])	1.004 (t(468150.467)=2.467, p=0.014, d=0.007, 95%CI=[1.001, 1.007])	0.974 (t(468182.764)=-12.199, p<0.001, d=0.036, 95%CI=[0.970, 0.978])	1.053 (t(468178.544)=26.419, p<0.001, d=0.077, 95%CI=[1.049, 1.057])	1.045 (t(468246.312)=22.118, p<0.001, d=0.065, 95%CI=[1.041, 1.049])
Positive	1.001 (t(468257.035)=0.918, p=0.359, d=0.003, 95%CI=[0.999, 1.004])	1.062 (t(468219.464)=34.743, p<0.001, d=0.102, 95%CI=[1.059, 1.066])	0.978 (t(468242.334)=-13.815, p<0.001, d=0.040, 95%CI=[0.975, 0.981])	1.057 (t(468259.162)=46.182, p<0.001, d=0.135, 95%CI=[1.054, 1.059])	0.953 (t(468215.096)=-44.146, p<0.001, d=0.129, 95%CI=[0.951, 0.955])	0.984 (t(468244.699)=-10.671, p<0.001, d=0.031, 95%CI=[0.981, 0.987])	0.942 (t(468212.519)=-44.100, p<0.001, d=0.129, 95%CI=[0.940, 0.945])	0.928 (t(468284.088)=-54.268, p<0.001, d=0.159, 95%CI=[0.926, 0.931])
Negative	1.004 (t(468287.195)=2.914, p=0.004, d=0.009, 95%CI=[1.001, 1.007])	0.991 (t(468228.794)=-5.134, p<0.001, d=0.015, 95%CI=[0.987, 0.994])	1.008 (t(468278.967)=4.865, p<0.001, d=0.014, 95%CI=[1.005, 1.011])	0.967 (t(468293.320)=-26.903, p<0.001, d=0.079, 95%CI=[0.965, 0.970])	1.016 (t(468268.757)=14.147, p<0.001, d=0.041, 95%CI=[1.014, 1.018])	0.977 (t(468288.365)=-15.116, p<0.001, d=0.044, 95%CI=[0.974, 0.980])	1.081 (t(468246.170)=55.392, p<0.001, d=0.162, 95%CI=[1.078, 1.084])	1.043 (t(468301.280)=29.483, p<0.001, d=0.086, 95%CI=[1.040, 1.046])
Total tokens	1.128 (t(468301.104)=43.647, p<0.001, d=0.128, 95%CI=[1.122, 1.134])	1.015 (t(468232.299)=4.057, p<0.001, d=0.012, 95%CI=[1.008, 1.022])	0.988 (t(468296.734)=-3.797, p<0.001, d=0.011, 95%CI=[0.981, 0.994])	0.982 (t(468290.118)=-7.489, p<0.001, d=0.022, 95%CI=[0.977, 0.986])	1.053 (t(468294.755)=22.998, p<0.001, d=0.067, 95%CI=[1.048, 1.057])	0.945 (t(468297.805)=-18.240, p<0.001, d=0.053, 95%CI=[0.940, 0.951])	1.046 (t(468261.053)=15.981, p<0.001, d=0.047, 95%CI=[1.040, 1.051])	1.035 (t(468181.480)=12.228, p<0.001, d=0.036, 95%CI=[1.029, 1.041])
Followers at posting	2.876 (t(413.677)=13.382, p<0.001, d=1.316, 95%CI=[2.464, 3.357])	443.015 (t(25454.113)=46.437, p<0.001, d=0.582, 95%CI=[342.548, 572.947])	6.907 (t(453.199)=19.601, p<0.001, d=1.841, 95%CI=[5.694, 8.380])	2.180 (t(171.265)=13.065, p<0.001, d=1.997, 95%CI=[1.940, 2.451])	0.654 (t(304.289)=-6.359, p<0.001, d=0.729, 95%CI=[0.574, 0.746])	3.401 (t(168.027)=15.499, p<0.001, d=2.391, 95%CI=[2.914, 3.971])	0.241 (t(1440.266)=-15.184, p<0.001, d=0.800, 95%CI=[0.200, 0.289])	1.903 (t(136.849)=11.970, p<0.001, d=2.046, 95%CI=[1.713, 2.115])
N	468310	468310	468310	468310	468310	468310	468310	468310
N (page)	84	84	84	84	84	84	84	84
AIC	1313391.005	1560140.339	1478598.776	1203259.373	1115045.250	1414372.056	1320624.197	1333765.981
BIC	1313501.574	1560250.907	1478709.345	1203369.942	1115155.819	1414482.625	1320734.766	1333876.550
R2 (fixed)	0.390	0.696	0.574	0.347	0.126	0.442	0.392	0.244
R2 (total)	0.614	0.964	0.754	0.499	0.493	0.593	0.780	0.340

Mixed-effects linear regression. DV is log-transformed engagement. Estimates are exponentiated. P-values estimated using Satterthwaite d.f. Cohen's d (d) estimated using effectsize::t_to_d(t,df_error). See the jtools package and Methods for more information.

3.19. Supplementary Table 19. Study 1: Predictors of Facebook reactions on Ukrainian social media before the invasion (pro-Russian).

	Share Pro-Russian Facebook Before	Like Pro-Russian Facebook Before	Comment Pro-Russian Facebook Before	Love Pro-Russian Facebook Before	Wow Pro-Russian Facebook Before	Haha Pro-Russian Facebook Before	Sad Pro-Russian Facebook Before	Angry Pro-Russian Facebook Before
(Intercept)	2.567 (t(6.683)=6.209, p=0.001, d=4.804, 95%CI=[1.906, 3.457])	15.647 (t(9.815)=10.519, p<0.001, d=6.715, 95%CI=[9.373, 26.121])	9.855 (t(10.870)=5.333, p<0.001, d=3.235, 95%CI=[4.251, 22.846])	2.076 (t(12.978)=10.478, p<0.001, d=5.817, 95%CI=[1.811, 2.380])	2.009 (t(7.916)=4.161, p=0.003, d=2.957, 95%CI=[1.446, 2.791])	4.367 (t(10.481)=4.428, p=0.001, d=2.736, 95%CI=[2.274, 8.386])	1.681 (t(8.182)=2.206, p=0.058, d=1.542, 95%CI=[1.060, 2.667])	2.447 (t(7.611)=4.609, p=0.002, d=3.341, 95%CI=[1.673, 3.580])
Ingroup mentions	0.988 (t(114504.133)=-4.850, p<0.001, d=0.029, 95%CI=[0.983, 0.993])	1.058 (t(114541.112)=17.435, p<0.001, d=0.103, 95%CI=[1.051, 1.065])	1.099 (t(114548.766)=27.571, p<0.001, d=0.163, 95%CI=[1.092, 1.107])	1.046 (t(114382.264)=19.686, p<0.001, d=0.116, 95%CI=[1.041, 1.051])	0.994 (t(114537.264)=-2.869, p=0.004, d=0.017, 95%CI=[0.990, 0.998])	1.109 (t(114548.991)=36.638, p<0.001, d=0.217, 95%CI=[1.103, 1.116])	0.962 (t(114545.860)=-15.243, p<0.001, d=0.090, 95%CI=[0.957, 0.967])	1.015 (t(114537.474)=5.982, p<0.001, d=0.035, 95%CI=[1.010, 1.020])
Outgroup mentions	1.031 (t(114131.948)=10.112, p<0.001, d=0.060, 95%CI=[1.025, 1.037])	1.093 (t(114416.373)=23.019, p<0.001, d=0.136, 95%CI=[1.085, 1.102])	1.204 (t(114531.434)=45.205, p<0.001, d=0.267, 95%CI=[1.194, 1.213])	1.033 (t(113775.963)=11.911, p<0.001, d=0.071, 95%CI=[1.028, 1.039])	1.056 (t(114368.507)=21.154, p<0.001, d=0.125, 95%CI=[1.050, 1.061])	1.225 (t(114522.620)=59.748, p<0.001, d=0.353, 95%CI=[1.217, 1.233])	1.001 (t(114451.432)=0.481, p=0.630, d=0.003, 95%CI=[0.996, 1.007])	1.067 (t(114366.794)=21.978, p<0.001, d=0.130, 95%CI=[1.061, 1.073])
Moral emotional	1.025 (t(114525.078)=7.079, p<0.001, d=0.042, 95%CI=[1.018, 1.032])	1.060 (t(114531.858)=13.343, p<0.001, d=0.079, 95%CI=[1.051, 1.069])	1.058 (t(114532.412)=12.157, p<0.001, d=0.072, 95%CI=[1.049, 1.068])	1.019 (t(114537.600)=5.996, p<0.001, d=0.035, 95%CI=[1.013, 1.025])	1.022 (t(114527.864)=7.439, p<0.001, d=0.044, 95%CI=[1.016, 1.028])	1.022 (t(114531.944)=5.686, p<0.001, d=0.034, 95%CI=[1.014, 1.030])	1.007 (t(114527.929)=2.129, p=0.033, d=0.013, 95%CI=[1.001, 1.014])	1.033 (t(114526.980)=9.639, p<0.001, d=0.057, 95%CI=[1.026, 1.039])
Positive	1.008 (t(114525.758)=3.316, p=0.001, d=0.020, 95%CI=[1.003, 1.012])	1.042 (t(114531.568)=14.158, p<0.001, d=0.084, 95%CI=[1.036, 1.048])	0.993 (t(114532.028)=-2.409, p=0.016, d=0.014, 95%CI=[0.987, 0.999])	1.050 (t(114542.454)=23.429, p<0.001, d=0.138, 95%CI=[1.045, 1.054])	0.975 (t(114527.558)=-13.265, p<0.001, d=0.078, 95%CI=[0.971, 0.978])	1.002 (t(114531.533)=0.817, p=0.414, d=0.005, 95%CI=[0.997, 1.007])	0.974 (t(114527.434)=-11.685, p<0.001, d=0.069, 95%CI=[0.969, 0.978])	0.968 (t(114526.642)=-14.903, p<0.001, d=0.088, 95%CI=[0.963, 0.972])
Negative	1.021 (t(114523.128)=8.130, p<0.001, d=0.048, 95%CI=[1.016, 1.026])	1.021 (t(114530.421)=6.364, p<0.001, d=0.038, 95%CI=[1.014, 1.027])	1.044 (t(114531.523)=12.752, p<0.001, d=0.075, 95%CI=[1.037, 1.051])	0.984 (t(114539.022)=-7.320, p<0.001, d=0.043, 95%CI=[0.979, 0.988])	1.033 (t(114526.067)=15.521, p<0.001, d=0.092, 95%CI=[1.029, 1.038])	0.999 (t(114530.949)=-0.403, p=0.687, d=0.002, 95%CI=[0.993, 1.004])	1.077 (t(114526.334)=29.626, p<0.001, d=0.175, 95%CI=[1.072, 1.082])	1.049 (t(114525.116)=19.650, p<0.001, d=0.116, 95%CI=[1.044, 1.054])
Total tokens	1.038 (t(114534.124)=6.028, p<0.001, d=0.036, 95%CI=[1.026, 1.051])	0.935 (t(114548.980)=-8.407, p<0.001, d=0.050, 95%CI=[0.921, 0.950])	0.909 (t(114542.873)=-11.332, p<0.001, d=0.067, 95%CI=[0.894, 0.924])	0.906 (t(114128.361)=-17.544, p<0.001, d=0.104, 95%CI=[0.896, 0.916])	1.026 (t(114548.982)=4.826, p<0.001, d=0.029, 95%CI=[1.015, 1.036])	0.911 (t(114543.781)=-13.327, p<0.001, d=0.079, 95%CI=[0.899, 0.924])	1.017 (t(114547.357)=2.786, p=0.005, d=0.016, 95%CI=[1.005, 1.030])	1.028 (t(114548.999)=4.577, p<0.001, d=0.027, 95%CI=[1.016, 1.040])
Followers at posting	0.963 (t(36.018)=-0.360, p=0.721, d=0.120, 95%CI=[0.783, 1.184])	4.902 (t(116.107)=10.602, p<0.001, d=1.968, 95%CI=[3.654, 6.576])	7.882 (t(523.311)=11.857, p<0.001, d=1.037, 95%CI=[5.603, 11.088])	1.557 (t(22.995)=6.962, p<0.001, d=2.904, 95%CI=[1.375, 1.764])	0.734 (t(85.761)=-3.143, p=0.002, d=0.679, 95%CI=[0.606, 0.890])	4.100 (t(405.927)=9.896, p<0.001, d=0.982, 95%CI=[3.101, 5.422])	0.521 (t(148.411)=-5.364, p<0.001, d=0.881, 95%CI=[0.410, 0.661])	2.393 (t(84.830)=7.725, p<0.001, d=1.677, 95%CI=[1.918, 2.987])
N	114557	114557	114557	114557	114557	114557	114557	114557
N (page)	15	15	15	15	15	15	15	15
AIC	356465.159	412363.342	425465.131	333372.761	317620.240	381908.941	355876.763	348935.174
BIC	356561.647	412459.830	425561.619	333469.249	317716.729	382005.429	355973.251	349031.662
R2 (fixed)	0.009	0.408	0.414	0.137	0.075	0.340	0.159	0.266
R2 (total)	0.212	0.598	0.726	0.190	0.359	0.671	0.484	0.495

Mixed-effects linear regression. DV is log-transformed engagement. Estimates are exponentiated. P-values estimated using Satterthwaite d.f. Cohen's d (d) estimated using effectsize::t_to_d(t,df_error). See the jtools package and Methods for more information.

3.20. Supplementary Table 20. Study 1: Predictors of Facebook reactions on Ukrainian social media after the invasion.

	Share Pro-Ukrainian Facebook After	Like Pro-Ukrainian Facebook After	Comment Pro-Ukrainian Facebook After	Love Pro-Ukrainian Facebook After	Wow Pro-Ukrainian Facebook After	Haha Pro-Ukrainian Facebook After	Sad Pro-Ukrainian Facebook After	Angry Pro-Ukrainian Facebook After
(Intercept)	1.990 (t(83.205)=2.909, p=0.005, d=0.638, 95%CI=[1.252, 3.163])	10.932 (t(83.202)=9.514, p<0.001, d=2.086, 95%CI=[6.679, 17.893])	3.089 (t(82.700)=8.112, p<0.001, d=1.784, 95%CI=[2.352, 4.057])	1.806 (t(82.467)=4.769, p<0.001, d=1.050, 95%CI=[1.416, 2.302])	1.511 (t(82.472)=10.200, p<0.001, d=2.246, 95%CI=[1.396, 1.636])	1.818 (t(83.967)=17.320, p<0.001, d=3.780, 95%CI=[1.699, 1.945])	1.629 (t(82.390)=4.674, p<0.001, d=1.030, 95%CI=[1.328, 2.000])	1.777 (t(82.073)=6.589, p<0.001, d=1.455, 95%CI=[1.498, 2.108])
Ingroup mentions	0.990 (t(535713.555)=-7.102, p<0.001, d=0.019, 95%CI=[0.988, 0.993])	1.063 (t(535717.444)=33.915, p<0.001, d=0.093, 95%CI=[1.060, 1.067])	1.058 (t(535729.522)=37.831, p<0.001, d=0.103, 95%CI=[1.055, 1.061])	1.039 (t(535741.220)=22.687, p<0.001, d=0.062, 95%CI=[1.035, 1.042])	0.986 (t(535778.221)=-16.202, p<0.001, d=0.044, 95%CI=[0.984, 0.987])	0.981 (t(535740.060)=-14.585, p<0.001, d=0.040, 95%CI=[0.979, 0.984])	0.935 (t(535750.589)=-41.148, p<0.001, d=0.112, 95%CI=[0.932, 0.938])	0.984 (t(535758.022)=-10.610, p<0.001, d=0.029, 95%CI=[0.981, 0.987])
Outgroup mentions	1.014 (t(535717.113)=9.708, p<0.001, d=0.027, 95%CI=[1.011, 1.017])	1.033 (t(535722.768)=16.865, p<0.001, d=0.046, 95%CI=[1.029, 1.037])	1.077 (t(535739.765)=46.979, p<0.001, d=0.128, 95%CI=[1.074, 1.080])	0.996 (t(535754.895)=-2.544, p=0.011, d=0.007, 95%CI=[0.992, 0.999])	1.029 (t(535788.578)=29.897, p<0.001, d=0.082, 95%CI=[1.027, 1.031])	1.166 (t(535603.707)=113.075, p<0.001, d=0.309, 95%CI=[1.162, 1.169])	0.891 (t(535765.935)=-66.827, p<0.001, d=0.183, 95%CI=[0.888, 0.894])	1.095 (t(535773.899)=57.322, p<0.001, d=0.157, 95%CI=[1.092, 1.099])
Moral emotional	1.034 (t(535707.654)=18.653, p<0.001, d=0.051, 95%CI=[1.030, 1.038])	1.027 (t(535708.415)=11.392, p<0.001, d=0.031, 95%CI=[1.023, 1.032])	1.005 (t(535710.504)=2.356, p=0.018, d=0.006, 95%CI=[1.001, 1.008])	1.033 (t(535712.848)=14.651, p<0.001, d=0.040, 95%CI=[1.028, 1.037])	0.981 (t(535723.121)=-15.973, p<0.001, d=0.044, 95%CI=[0.979, 0.984])	0.964 (t(535747.206)=-21.901, p<0.001, d=0.060, 95%CI=[0.961, 0.967])	1.081 (t(535714.942)=36.612, p<0.001, d=0.100, 95%CI=[1.077, 1.086])	1.013 (t(535716.537)=6.799, p<0.001, d=0.019, 95%CI=[1.010, 1.017])
Positive	1.005 (t(535708.799)=3.189, p=0.001, d=0.009, 95%CI=[1.002, 1.008])	1.090 (t(535710.195)=43.755, p<0.001, d=0.120, 95%CI=[1.085, 1.094])	1.013 (t(535714.423)=7.732, p<0.001, d=0.021, 95%CI=[1.009, 1.016])	1.070 (t(535718.951)=37.157, p<0.001, d=0.102, 95%CI=[1.066, 1.073])	0.976 (t(535737.700)=-25.253, p<0.001, d=0.069, 95%CI=[0.974, 0.978])	0.999 (t(535773.283)=-0.505, p=0.614, d=0.001, 95%CI=[0.997, 1.002])	0.925 (t(535722.903)=-44.164, p<0.001, d=0.121, 95%CI=[0.922, 0.928])	0.905 (t(535726.124)=-61.158, p<0.001, d=0.167, 95%CI=[0.902, 0.908])
Negative	0.993 (t(535713.295)=-5.207, p<0.001, d=0.014, 95%CI=[0.991, 0.996])	0.998 (t(535717.103)=-1.201, p=0.230, d=0.003, 95%CI=[0.995, 1.001])	0.984 (t(535729.081)=-11.708, p<0.001, d=0.032, 95%CI=[0.981, 0.986])	0.987 (t(535740.848)=-8.023, p<0.001, d=0.022, 95%CI=[0.984, 0.990])	0.989 (t(535778.562)=-13.198, p<0.001, d=0.036, 95%CI=[0.987, 0.990])	0.968 (t(535730.110)=-26.425, p<0.001, d=0.072, 95%CI=[0.966, 0.971])	1.017 (t(535750.350)=10.806, p<0.001, d=0.030, 95%CI=[1.014, 1.020])	1.017 (t(535757.927)=11.761, p<0.001, d=0.032, 95%CI=[1.014, 1.020])
Total tokens	1.065 (t(535739.691)=24.022, p<0.001, d=0.066, 95%CI=[1.060, 1.071])	0.924 (t(535754.656)=-22.475, p<0.001, d=0.061, 95%CI=[0.918, 0.931])	0.989 (t(535785.433)=-3.817, p<0.001, d=0.010, 95%CI=[0.983, 0.995])	0.903 (t(535783.457)=-31.622, p<0.001, d=0.086, 95%CI=[0.897, 0.909])	1.049 (t(535318.180)=27.924, p<0.001, d=0.076, 95%CI=[1.046, 1.053])	0.995 (t(529836.762)=-2.159, p=0.031, d=0.006, 95%CI=[0.990, 1.000])	1.160 (t(535751.183)=47.159, p<0.001, d=0.129, 95%CI=[1.153, 1.167])	1.028 (t(535697.193)=9.503, p<0.001, d=0.026, 95%CI=[1.022, 1.034])
Followers at posting	0.196 (t(528101.209)=-128.771, p<0.001, d=0.354, 95%CI=[0.191, 0.201])	0.343 (t(516138.587)=-63.737, p<0.001, d=0.177, 95%CI=[0.332, 0.355])	0.756 (t(445179.339)=-20.215, p<0.001, d=0.061, 95%CI=[0.736, 0.777])	0.550 (t(351634.038)=-38.618, p<0.001, d=0.130, 95%CI=[0.534, 0.567])	1.097 (t(110903.763)=11.298, p<0.001, d=0.068, 95%CI=[1.080, 1.115])	1.367 (t(17725.943)=27.188, p<0.001, d=0.408, 95%CI=[1.336, 1.398])	0.657 (t(276921.924)=-28.029, p<0.001, d=0.107, 95%CI=[0.638, 0.676])	0.753 (t(222361.259)=-20.531, p<0.001, d=0.087, 95%CI=[0.733, 0.773])
N	535797	535797	535797	535797	535797	535797	535797	535797
N (page)	84	84	84	84	84	84	84	84
AIC	1667622.319	1970132.592	1762927.823	1885875.611	1214909.339	1599419.772	1855392.288	1770052.175
BIC	1667734.234	1970244.508	1763039.738	1885987.527	1215021.254	1599531.687	1855504.203	1770164.090
R2 (fixed)	0.331	0.146	0.032	0.111	0.018	0.101	0.077	0.051
R2 (total)	0.853	0.740	0.521	0.459	0.206	0.169	0.377	0.319

Mixed-effects linear regression. DV is log-transformed engagement. Estimates are exponentiated. P-values estimated using Satterthwaite d.f. Cohen's d (d) estimated using effectsize::t_to_d(t,df_error). See the jtools package and Methods for more information.

3.21. Supplementary Table 21. Predictors of engagement on Ukrainian social media after the invasion without identity mentions variables.

	Pro-Ukrainian Twitter Before	Pro-Ukrainian Twitter After	Pro-Ukrainian Facebook Before	Pro-Ukrainian Facebook After	Pro-Ukrainian Geolocated Twitter Before	Pro-Ukrainian Geolocated Twitter After
(Intercept)	3.817 (t(62.370)=11.137, p<0.001, d=2.820, 95%CI=[3.016, 4.832])	2.514 (t(55.632)=1.622, p=0.110, d=0.435, 95%CI=[0.825, 7.656])	59.985 (t(77.174)=13.310, p<0.001, d=3.030, 95%CI=[32.825, 109.617])	34.660 (t(84.176)=14.680, p<0.001, d=3.200, 95%CI=[21.590, 55.644])	2.170 (t(7332.525)=35.817, p<0.001, d=0.837, 95%CI=[2.080, 2.264])	2.221 (t(9643.575)=49.311, p<0.001, d=1.004, 95%CI=[2.152, 2.292])
Ingroup solidarity	1.610 (t(180301.065)=65.057, p<0.001, d=0.306, 95%CI=[1.588, 1.634])	1.699 (t(217168.367)=100.986, p<0.001, d=0.433, 95%CI=[1.681, 1.716])	1.717 (t(465891.523)=95.927, p<0.001, d=0.281, 95%CI=[1.698, 1.736])	1.885 (t(535709.325)=151.540, p<0.001, d=0.414, 95%CI=[1.870, 1.901])	1.185 (t(134345.570)=20.124, p<0.001, d=0.110, 95%CI=[1.166, 1.205])	1.164 (t(147282.739)=22.587, p<0.001, d=0.118, 95%CI=[1.149, 1.180])
Outgroup hostility	1.325 (t(180310.252)=33.271, p<0.001, d=0.157, 95%CI=[1.303, 1.347])	1.039 (t(217168.866)=7.460, p<0.001, d=0.032, 95%CI=[1.029, 1.050])	1.344 (t(465971.899)=45.579, p<0.001, d=0.134, 95%CI=[1.327, 1.361])	1.084 (t(535710.131)=20.050, p<0.001, d=0.055, 95%CI=[1.076, 1.093])	1.157 (t(133208.010)=11.652, p<0.001, d=0.064, 95%CI=[1.129, 1.186])	1.077 (t(146438.010)=10.702, p<0.001, d=0.056, 95%CI=[1.062, 1.091])
Moral emotional	1.028 (t(180300.632)=6.417, p<0.001, d=0.030, 95%CI=[1.020, 1.037])	1.018 (t(217168.110)=4.546, p<0.001, d=0.020, 95%CI=[1.010, 1.025])	1.045 (t(465877.520)=17.260, p<0.001, d=0.051, 95%CI=[1.040, 1.050])	1.019 (t(535706.115)=8.893, p<0.001, d=0.024, 95%CI=[1.014, 1.023])	1.016 (t(133138.380)=3.469, p=0.001, d=0.019, 95%CI=[1.007, 1.026])	1.017 (t(146132.527)=3.715, p<0.001, d=0.019, 95%CI=[1.008, 1.025])
Positive	1.000 (t(180307.200)=-0.047, p=0.963, d=0.000, 95%CI=[0.993, 1.006])	1.010 (t(217167.622)=2.956, p=0.003, d=0.013, 95%CI=[1.003, 1.017])	0.983 (t(465891.633)=-9.587, p<0.001, d=0.028, 95%CI=[0.980, 0.986])	0.985 (t(535707.812)=-8.556, p<0.001, d=0.023, 95%CI=[0.982, 0.989])	1.014 (t(133637.537)=5.388, p<0.001, d=0.029, 95%CI=[1.009, 1.019])	1.002 (t(146371.650)=0.603, p=0.547, d=0.003, 95%CI=[0.996, 1.007])
Negative	1.030 (t(180309.696)=9.109, p<0.001, d=0.043, 95%CI=[1.024, 1.037])	1.005 (t(217168.594)=1.838, p=0.066, d=0.008, 95%CI=[1.000, 1.011])	1.023 (t(465902.734)=12.466, p<0.001, d=0.037, 95%CI=[1.020, 1.027])	1.001 (t(535713.962)=0.509, p=0.611, d=0.001, 95%CI=[0.998, 1.004])	0.999 (t(132833.554)=-0.176, p=0.861, d=0.001, 95%CI=[0.992, 1.006])	0.986 (t(145583.963)=-4.289, p<0.001, d=0.022, 95%CI=[0.980, 0.992])
Total tokens	1.112 (t(180358.622)=32.185, p<0.001, d=0.152, 95%CI=[1.105, 1.120])	1.073 (t(217189.325)=23.925, p<0.001, d=0.103, 95%CI=[1.067, 1.079])	1.024 (t(465888.104)=6.727, p<0.001, d=0.020, 95%CI=[1.017, 1.031])	0.964 (t(535747.099)=-12.860, p<0.001, d=0.035, 95%CI=[0.959, 0.969])	1.071 (t(133990.782)=26.220, p<0.001, d=0.143, 95%CI=[1.065, 1.076])	1.088 (t(146569.869)=29.648, p<0.001, d=0.155, 95%CI=[1.082, 1.094])
Followers count	2.373 (t(6983.183)=17.615, p<0.001, d=0.422, 95%CI=[2.155, 2.612])	0.017 (t(12728.101)=-24.564, p<0.001, d=0.435, 95%CI=[0.012, 0.023])			1.180 (t(6157.019)=9.564, p<0.001, d=0.244, 95%CI=[1.141, 1.221])	1.029 (t(14904.029)=7.347, p<0.001, d=0.120, 95%CI=[1.021, 1.037])
Followers at posting			154.269 (t(9726.493)=38.429, p<0.001, d=0.779, 95%CI=[119.309, 199.473])	0.458 (t(522530.897)=-53.332, p<0.001, d=0.148, 95%CI=[0.445, 0.471])		
Has media			1.378 (t(465884.851)=6.630, p<0.001, d=0.019, 95%CI=[1.253, 1.515])	0.852 (t(535713.009)=-8.134, p<0.001, d=0.022, 95%CI=[0.820, 0.886])		
Has URL			0.673 (t(465882.624)=-8.271, p<0.001, d=0.024, 95%CI=[0.613, 0.739])	0.422 (t(535712.616)=-44.654, p<0.001, d=0.122, 95%CI=[0.407, 0.439])	1.135 (t(136099.345)=23.415, p<0.001, d=0.127, 95%CI=[1.123, 1.147])	1.000 (t(147568.598)=0.042, p=0.966, d=0.000, 95%CI=[0.989, 1.011])
User is not verified					0.894 (t(6889.138)=-4.282, p<0.001, d=0.103, 95%CI=[0.850, 0.941])	1.005 (t(8024.049)=0.208, p=0.835, d=0.005, 95%CI=[0.957, 1.056])
User is verified					3.013 (t(5962.324)=10.057, p<0.001, d=0.260, 95%CI=[2.430, 3.736])	7.286 (t(7126.597)=16.457, p<0.001, d=0.390, 95%CI=[5.751, 9.230])
N	180367	217245	465982	535797	136950	149230
N (accounts)	63	63	84	84	6446	7596
AIC	452828.967	598672.293	1561941.306	1820104.672	308555.639	371302.371
BIC	452929.994	598775.181	1562073.929	1820238.970	308683.395	371431.243
R2 (fixed)	0.308	0.464	0.702	0.112	0.040	0.051
R2 (total)	0.692	0.977	0.947	0.765	0.614	0.593

Mixed-effects linear regression. DV is log-transformed engagement. Estimates are exponentiated. P-values estimated using Satterthwaite d.f. Cohen's d (d) estimated using effectsize::t_to_d(t,df_error). See the jtools package and Methods for more information.

3.22. Supplementary Table 22. Study 3: Predictors of engagement on geolocated Ukrainian Twitter before and after the invasion (only posts where full user data is available).

	Pro-Ukrainian Geolocated Twitter Before (Full)	Pro-Ukrainian Geolocated Twitter After (Full)
(Intercept)	1.922 (t(5332.247)=42.207, p<0.001, d=1.156, 95%CI=[1.864, 1.981])	2.270 (t(3606.285)=39.141, p<0.001, d=1.304, 95%CI=[2.179, 2.365])
Ingroup mentions (binary)	1.066 (t(93687.258)=7.544, p<0.001, d=0.049, 95%CI=[1.048, 1.084])	1.081 (t(76443.034)=8.353, p<0.001, d=0.060, 95%CI=[1.061, 1.101])
Outgroup mentions (binary)	1.087 (t(92589.609)=4.620, p<0.001, d=0.030, 95%CI=[1.049, 1.125])	1.057 (t(75713.785)=4.614, p<0.001, d=0.034, 95%CI=[1.033, 1.083])
Ingroup solidarity	1.313 (t(93309.366)=17.002, p<0.001, d=0.111, 95%CI=[1.272, 1.355])	1.172 (t(76348.818)=12.190, p<0.001, d=0.088, 95%CI=[1.143, 1.202])
Outgroup hostility	1.110 (t(92003.537)=4.866, p<0.001, d=0.032, 95%CI=[1.064, 1.157])	1.063 (t(75595.134)=5.205, p<0.001, d=0.038, 95%CI=[1.039, 1.088])
Positive	1.015 (t(92542.258)=4.523, p<0.001, d=0.030, 95%CI=[1.008, 1.021])	1.000 (t(75941.393)=0.026, p=0.979, d=0.000, 95%CI=[0.992, 1.009])
Negative	0.996 (t(91977.633)=-0.916, p=0.360, d=0.006, 95%CI=[0.987, 1.005])	0.988 (t(75605.494)=-2.520, p=0.012, d=0.018, 95%CI=[0.979, 0.997])
Moral emotional	1.021 (t(92156.176)=3.492, p<0.001, d=0.023, 95%CI=[1.009, 1.033])	1.015 (t(75778.424)=2.227, p=0.026, d=0.016, 95%CI=[1.002, 1.028])
Total tokens	1.071 (t(92711.991)=21.147, p<0.001, d=0.139, 95%CI=[1.064, 1.078])	1.106 (t(75999.072)=23.840, p<0.001, d=0.173, 95%CI=[1.097, 1.115])
Followers count	1.177 (t(4165.456)=9.490, p<0.001, d=0.294, 95%CI=[1.138, 1.217])	1.030 (t(6274.796)=7.302, p<0.001, d=0.184, 95%CI=[1.022, 1.038])
User is verified	3.329 (t(3995.688)=11.148, p<0.001, d=0.353, 95%CI=[2.695, 4.113])	7.089 (t(2760.109)=15.842, p<0.001, d=0.603, 95%CI=[5.564, 9.033])
Has URL	1.140 (t(94301.330)=19.712, p<0.001, d=0.128, 95%CI=[1.125, 1.155])	0.971 (t(76551.632)=-3.636, p<0.001, d=0.026, 95%CI=[0.956, 0.987])
N	94795	77251
N (author_id)	4332	2922
AIC	217608.544	201717.918
BIC	217740.977	201847.485
R2 (fixed)	0.052	0.082
R2 (total)	0.601	0.577

Mixed-effects linear regression. DV is log-transformed engagement. Estimates are exponentiated. P-values estimated using Satterthwaite d.f. Cohen’s d (d) estimated using effectsize::t_to_d(t,df_error). See the jtools package and Methods for more information.

3.23. Supplementary Table 23. Study 1: Predictors of engagement on geolocated Ukrainian Twitter before and after the invasion.

Full means the original ingroup and outgroup mentions dictionaries, W/O Cities means the dictionaries were without cities but including capitals (Kyiv and Moscow), W/O Cities and Politicians means the dictionaries were without cities but including capitals (Kyiv and Moscow) and without politicians, Just Country means the dictionaries were only composed of the name of the country (Ukraine and Russia).

	Pro-Ukrainian Twitter Before	Pro-Russian Twitter Before	Pro-Ukrainian Facebook Before	Pro-Russian Facebook Before	Pro-Ukrainian Twitter After	Pro-Ukrainian Facebook After
	Full	Full	Full	Full	Full	Full
Ingroup mentions	1.156 (t(182031.995)=46.658, p<0.001, d=0.219, 95%CI=[1.149, 1.163])	1.041 (t(46676.896)=6.491, p<0.001, d=0.060, 95%CI=[1.028, 1.053])	1.113 (t(468248.478)=62.266, p<0.001, d=0.182, 95%CI=[1.109, 1.117])	1.071 (t(114542.360)=20.242, p<0.001, d=0.120, 95%CI=[1.064, 1.079])	1.113 (t(217179.930)=35.993 , p<0.001, d=0.154, 95%CI=[1.107, 1.120])	1.043 (t(535713.511)=26.272 , p<0.001, d=0.072, 95%CI=[1.040, 1.046])
Outgroup mentions	1.229 (t(182021.378)=44.930, p<0.001, d=0.211, 95%CI=[1.218, 1.240])	1.035 (t(46680.324)=4.142, p<0.001, d=0.038, 95%CI=[1.018, 1.052])	1.160 (t(466964.379)=63.890, p<0.001, d=0.187, 95%CI=[1.154, 1.165])	1.184 (t(114461.159)=41.373, p<0.001, d=0.245, 95%CI=[1.175, 1.194])	1.056 (t(217172.191)=17.364 , p<0.001, d=0.075, 95%CI=[1.050, 1.063])	1.074 (t(535718.970)=41.712 , p<0.001, d=0.114, 95%CI=[1.070, 1.077])
	W/O Cities	W/O Cities	W/O Cities	W/O Cities	W/O Cities	W/O Cities
Ingroup mentions	1.176 (t(182003.907)=49.848, p<0.001, d=0.234, 95%CI=[1.168, 1.183])	1.038 (t(46676.439)=5.842, p<0.001, d=0.054, 95%CI=[1.025, 1.051])	1.127 (t(468245.068)=66.794, p<0.001, d=0.195, 95%CI=[1.123, 1.131])	1.070 (t(114541.851)=19.525, p<0.001, d=0.115, 95%CI=[1.063, 1.078])	1.153 (t(217198.722)=44.685 , p<0.001, d=0.192, 95%CI=[1.145, 1.160])	1.063 (t(535714.559)=35.592 , p<0.001, d=0.097, 95%CI=[1.059, 1.067])
Outgroup mentions	1.224 (t(182021.416)=43.738, p<0.001, d=0.205, 95%CI=[1.213, 1.235])	1.037 (t(46680.166)=4.296, p<0.001, d=0.040, 95%CI=[1.020, 1.055])	1.155 (t(466955.415)=61.379, p<0.001, d=0.180, 95%CI=[1.149, 1.160])	1.194 (t(114475.617)=42.396, p<0.001, d=0.251, 95%CI=[1.184, 1.203])	1.055 (t(217171.980)=16.824 , p<0.001, d=0.072, 95%CI=[1.048, 1.061])	1.071 (t(535719.041)=40.403 , p<0.001, d=0.110, 95%CI=[1.068, 1.075])
	W/O Cities and Politicians	W/O Cities and Politicians	W/O Cities and Politicians	W/O Cities and Politicians	W/O Cities and Politicians	W/O Cities and Politicians
Ingroup mentions	1.151 (t(182004.858)=38.077, p<0.001, d=0.179, 95%CI=[1.142, 1.159])	1.028 (t(46675.963)=4.143, p<0.001, d=0.038, 95%CI=[1.015, 1.041])	1.074 (t(468238.018)=34.631, p<0.001, d=0.101, 95%CI=[1.070, 1.078])	1.055 (t(114546.616)=13.500, p<0.001, d=0.080, 95%CI=[1.046, 1.063])	1.151 (t(217194.692)=40.306 , p<0.001, d=0.173, 95%CI=[1.143, 1.159])	1.052 (t(535714.663)=26.841 , p<0.001, d=0.073, 95%CI=[1.048, 1.056])
Outgroup mentions	1.271 (t(182010.654)=45.340, p<0.001, d=0.213, 95%CI=[1.257, 1.284])	1.030 (t(46679.433)=2.824, p=0.005, d=0.026, 95%CI=[1.009, 1.051])	1.172 (t(467585.912)=57.700, p<0.001, d=0.169, 95%CI=[1.166, 1.179])	1.148 (t(114477.424)=27.614, p<0.001, d=0.163, 95%CI=[1.137, 1.160])	1.062 (t(217170.370)=17.874 , p<0.001, d=0.077, 95%CI=[1.055, 1.069])	1.068 (t(535717.527)=34.744 , p<0.001, d=0.095, 95%CI=[1.064, 1.072])
	Just Country	Just Country	Just Country	Just Country	Just Country	Just Country
Ingroup mentions	1.188 (t(182004.642)=42.158, p<0.001, d=0.198, 95%CI=[1.178, 1.197])	1.062 (t(46677.002)=7.726, p<0.001, d=0.072, 95%CI=[1.046, 1.079])	1.077 (t(468246.163)=33.202, p<0.001, d=0.097, 95%CI=[1.072, 1.082])	1.068 (t(114546.580)=14.345, p<0.001, d=0.085, 95%CI=[1.058, 1.077])	1.169 (t(217183.284)=42.224 , p<0.001, d=0.181, 95%CI=[1.160, 1.177])	1.059 (t(535712.739)=28.561 , p<0.001, d=0.078, 95%CI=[1.055, 1.063])
Outgroup mentions	1.300 (t(182006.252)=45.733, p<0.001, d=0.214, 95%CI=[1.285, 1.314])	1.043 (t(46680.556)=3.482, p<0.001, d=0.032, 95%CI=[1.018, 1.067])	1.189 (t(467646.784)=56.208, p<0.001, d=0.164, 95%CI=[1.182, 1.196])	1.178 (t(114503.542)=29.147, p<0.001, d=0.172, 95%CI=[1.166, 1.192])	1.067 (t(217169.382)=18.411 , p<0.001, d=0.079, 95%CI=[1.059, 1.074])	1.067 (t(535717.490)=32.312 , p<0.001, d=0.088, 95%CI=[1.062, 1.071])
	Just Country	Just Country	Just Country	Just Country	Just Country	Just Country
Ingroup mentions	1.156 (t(182031.995)=46.659, p<0.001, d=0.219, 95%CI=[1.149, 1.163])	1.041 (t(46676.895)=6.493, p<0.001, d=0.060, 95%CI=[1.028, 1.053])	1.113 (t(468248.618)=62.119, p<0.001, d=0.182, 95%CI=[1.109, 1.116])	1.071 (t(114542.368)=20.127, p<0.001, d=0.119, 95%CI=[1.064, 1.078])	1.113 (t(217179.922)=35.984 , p<0.001, d=0.154, 95%CI=[1.107, 1.120])	1.043 (t(535713.509)=26.206 , p<0.001, d=0.072, 95%CI=[1.040, 1.046])
Outgroup mentions	1.229 (t(182021.367)=44.927, p<0.001, d=0.211, 95%CI=[1.218, 1.240])	1.035 (t(46680.324)=4.140, p<0.001, d=0.038, 95%CI=[1.018, 1.052])	1.160 (t(466963.808)=63.856, p<0.001, d=0.187, 95%CI=[1.154, 1.165])	1.184 (t(114461.018)=41.297, p<0.001, d=0.244, 95%CI=[1.174, 1.193])	1.056 (t(217172.192)=17.369 , p<0.001, d=0.075, 95%CI=[1.050, 1.063])	1.074 (t(535718.965)=41.705 , p<0.001, d=0.114, 95%CI=[1.070, 1.077])

3.24. Supplementary Table 24. Study 1 Variance Inflation Factors.

Term	VIF	VIF_CI_low	VIF_CI_high	Model
ingroup mentions	1.02292840670341	1.01859437679352	1.02827262455714	Pro-Ukrainian Twitter Before
outgroup mentions	1.02995890128481	1.02546540829465	1.03524529258704	Pro-Ukrainian Twitter Before
moral-emotional	1.20426213486278	1.19789379286988	1.21083541395426	Pro-Ukrainian Twitter Before
positive	1.08705982337383	1.08183042133469	1.09262341220121	Pro-Ukrainian Twitter Before
negative	1.20695810714075	1.20056445515655	1.21355557782086	Pro-Ukrainian Twitter Before
total tokens	1.15033389359283	1.14447673206486	1.15642850748199	Pro-Ukrainian Twitter Before
follower count	1.00155400112433	1.00008012863469	1.0301380337239	Pro-Ukrainian Twitter Before
is retweet	1.02026408998438	1.01600647794811	1.02565419727101	Pro-Ukrainian Twitter Before
ingroup mentions	1.01000951299443	1.00397320275154	1.02521652094067	Pro-Russian Twitter Before
outgroup mentions	1.01329521827772	1.00660153786504	1.02677600775239	Pro-Russian Twitter Before
moral-emotional	1.16021298847108	1.14867718457865	1.17264385082066	Pro-Russian Twitter Before
positive	1.11344012887357	1.10284412713274	1.1251278337191	Pro-Russian Twitter Before
negative	1.15475374336237	1.14332459835162	1.16709428360589	Pro-Russian Twitter Before
total tokens	1.15029318514717	1.13895163946263	1.16256045332776	Pro-Russian Twitter Before
follower count	1.00035396774487	1	48009632.2383673	Pro-Russian Twitter Before
is retweet	1.00168597454858	1.00000764550727	1.37178830352302	Pro-Russian Twitter Before
ingroup mentions	1.03101783091647	1.02685871318296	1.03582099515375	Pro-Ukrainian Twitter After
outgroup mentions	1.06675789934449	1.06215780719168	1.07169842898649	Pro-Ukrainian Twitter After
moral-emotional	1.39546254860144	1.38800322960478	1.40306527217736	Pro-Ukrainian Twitter After
positive	1.08308037295231	1.0783187466856	1.08813149676161	Pro-Ukrainian Twitter After
negative	1.45163549937394	1.44370302500686	1.459709789654	Pro-Ukrainian Twitter After
total tokens	1.16564229827995	1.16013798261138	1.17133581010601	Pro-Ukrainian Twitter After
follower count	1.00145011113592	1.00007914752321	1.02656839053662	Pro-Ukrainian Twitter After
is retweet	1.01024150512222	1.00673602271464	1.01557126981481	Pro-Ukrainian Twitter After
ingroup mentions	1.12197154770048	1.11846044806931	1.12558671430777	Pro-Ukrainian Facebook Before
outgroup mentions	1.05745248971616	1.0543465355914	1.06073595195473	Pro-Ukrainian Facebook Before
moral-emotional	1.34194867023602	1.33715987271777	1.34680548469082	Pro-Ukrainian Facebook Before
positive	1.32432693707358	1.31963943178682	1.329083184523	Pro-Ukrainian Facebook Before
negative	1.37019853420317	1.36524748008591	1.37521670154697	Pro-Ukrainian Facebook Before
total tokens	1.67952677484633	1.67280397226859	1.68631675311915	Pro-Ukrainian Facebook Before
followers at posting	1.00829442930242	1.00583904199925	1.01178233646232	Pro-Ukrainian Facebook Before
has media	39.7891420559957	39.5647471002126	40.0148426885942	Pro-Ukrainian Facebook Before
has URL	39.7342190154798	39.5101377574009	39.9596041486719	Pro-Ukrainian Facebook Before
ingroup mentions	1.12437908753328	1.11735473962086	1.1318238825768	Pro-Russian Facebook Before
outgroup mentions	1.05623764333354	1.05015144898902	1.06306243571154	Pro-Russian Facebook Before
moral-emotional	1.50256766765219	1.4910907089604	1.51431284681407	Pro-Russian Facebook Before
positive	1.74182924067726	1.72758697618671	1.75635029259043	Pro-Russian Facebook Before
negative	1.56221859147771	1.55005136857551	1.57465495526676	Pro-Russian Facebook Before
total tokens	2.30926349351113	2.28847842877282	2.3303838521174	Pro-Russian Facebook Before
followers at posting	1.00558016558463	1.00195425194348	1.01593358934901	Pro-Russian Facebook Before
has media	17.9718974119833	17.7707688213576	18.1754380989414	Pro-Russian Facebook Before
has URL	17.947366374038	17.746520223133	18.150621334403	Pro-Russian Facebook Before
ingroup mentions	1.20058910204767	1.19687224292289	1.20437613379583	Pro-Ukrainian Facebook After
outgroup mentions	1.17777900499169	1.1741862289722	1.18144588583338	Pro-Ukrainian Facebook After
moral-emotional	1.74886756082628	1.74220952089645	1.75558532714664	Pro-Ukrainian Facebook After
positive	1.45754357688652	1.45244445912929	1.46270016247516	Pro-Ukrainian Facebook After
negative	1.90016400662932	1.8926971256805	1.9076933435999	Pro-Ukrainian Facebook After
total tokens	2.13117532356772	2.12247385225029	2.13994424910953	Pro-Ukrainian Facebook After
followers at posting	1.00975974730318	1.00737846054055	1.01290955842325	Pro-Ukrainian Facebook After
has media	9.58927127500054	9.54073494611416	9.63808343204888	Pro-Ukrainian Facebook After
has URL	9.66511566330802	9.61617426185039	9.71433506062599	Pro-Ukrainian Facebook After

3.25. Supplementary Table 25. Study 2: Predictors of Twitter reactions on Ukrainian social media before and after the invasion.

	RT Pro-Ukrainian Twitter Before	Favorite Pro-Ukrainian Twitter Before	RT Pro-Ukrainian Twitter After	Favorite Pro-Ukrainian Twitter After
(Intercept)	1.958 (t(62.537)=8.520, p<0.001, d=2.155, 95%CI=[1.677, 2.285])	2.750 (t(62.612)=9.038, p<0.001, d=2.284, 95%CI=[2.209, 3.425])	0.628 (t(59.008)=-0.611, p=0.543, d=0.159, 95%CI=[0.142, 2.788])	1.696 (t(51.989)=1.081, p=0.285, d=0.300, 95%CI=[0.651, 4.423])
Ingroup mentions (binary)	1.116 (t(180325.608)=29.217, p<0.001, d=0.138, 95%CI=[1.108, 1.125])	1.157 (t(180317.110)=33.663, p<0.001, d=0.159, 95%CI=[1.147, 1.167])	1.046 (t(214814.763)=11.955, p<0.001, d=0.052, 95%CI=[1.038, 1.054])	1.084 (t(214825.265)=17.332, p<0.001, d=0.075, 95%CI=[1.074, 1.094])
Outgroup mentions (binary)	1.269 (t(180351.414)=40.013, p<0.001, d=0.188, 95%CI=[1.254, 1.284])	1.335 (t(180341.470)=42.245, p<0.001, d=0.199, 95%CI=[1.317, 1.353])	1.126 (t(214809.109)=30.323, p<0.001, d=0.131, 95%CI=[1.118, 1.135])	1.155 (t(214806.229)=29.468, p<0.001, d=0.127, 95%CI=[1.144, 1.166])
Ingroup solidarity	1.220 (t(180301.725)=30.561, p<0.001, d=0.144, 95%CI=[1.205, 1.236])	1.505 (t(180299.860)=54.639, p<0.001, d=0.257, 95%CI=[1.483, 1.527])	1.252 (t(214806.919)=48.621, p<0.001, d=0.210, 95%CI=[1.241, 1.264])	1.770 (t(214798.286)=99.042, p<0.001, d=0.427, 95%CI=[1.750, 1.790])
Outgroup hostility	1.158 (t(180298.536)=18.298, p<0.001, d=0.086, 95%CI=[1.140, 1.177])	1.045 (t(180297.439)=4.768, p<0.001, d=0.022, 95%CI=[1.026, 1.064])	1.115 (t(214807.063)=23.618, p<0.001, d=0.102, 95%CI=[1.105, 1.125])	0.922 (t(214798.848)=-14.168, p<0.001, d=0.061, 95%CI=[0.911, 0.932])
Moral emotional	1.040 (t(180301.059)=10.323, p<0.001, d=0.049, 95%CI=[1.032, 1.048])	1.014 (t(180299.141)=3.103, p=0.002, d=0.015, 95%CI=[1.005, 1.022])	1.016 (t(214806.952)=4.977, p<0.001, d=0.021, 95%CI=[1.010, 1.023])	1.014 (t(214798.365)=3.404, p=0.001, d=0.015, 95%CI=[1.006, 1.022])
Positive	0.981 (t(180309.113)=-6.833, p<0.001, d=0.032, 95%CI=[0.975, 0.986])	1.007 (t(180306.029)=2.262, p=0.024, d=0.011, 95%CI=[1.001, 1.014])	0.990 (t(214806.759)=-3.475, p=0.001, d=0.015, 95%CI=[0.984, 0.996])	1.004 (t(214797.626)=1.111, p=0.267, d=0.005, 95%CI=[0.997, 1.011])
Negative	1.043 (t(180315.817)=14.793, p<0.001, d=0.070, 95%CI=[1.037, 1.048])	1.012 (t(180309.293)=3.753, p<0.001, d=0.018, 95%CI=[1.006, 1.019])	1.000 (t(214806.925)=0.060, p=0.952, d=0.000, 95%CI=[0.995, 1.005])	0.996 (t(214798.286)=-1.307, p=0.191, d=0.006, 95%CI=[0.990, 1.002])
Total tokens	1.126 (t(180309.070)=41.191, p<0.001, d=0.194, 95%CI=[1.119, 1.132])	0.999 (t(180353.583)=-0.230, p=0.818, d=0.001, 95%CI=[0.993, 1.006])	1.122 (t(214814.613)=45.337, p<0.001, d=0.196, 95%CI=[1.116, 1.127])	0.972 (t(214825.887)=-8.901, p<0.001, d=0.038, 95%CI=[0.966, 0.978])
Followers count	1.683 (t(2629.817)=12.686, p<0.001, d=0.495, 95%CI=[1.553, 1.824])	2.201 (t(5561.722)=16.300, p<0.001, d=0.437, 95%CI=[2.002, 2.420])	0.002 (t(56895.861)=-42.990, p<0.001, d=0.360, 95%CI=[0.001, 0.002])	0.033 (t(5257.871)=-19.321, p<0.001, d=0.533, 95%CI=[0.023, 0.047])
N	180367	180367	214882	214882
N (user_id)	63	63	63	63
AIC	400227.721	449852.572	523489.097	618281.438
BIC	400348.954	449973.805	523612.431	618404.772
R2 (fixed)	0.241	0.282	0.540	0.443
R2 (total)	0.557	0.658	0.992	0.964

Mixed-effects linear regression. DV is log-transformed engagement. Estimates are exponentiated. P-values estimated using Satterthwaite d.f. Cohen's d (d) estimated using effectsize::t_to_d(t,df_error). See the jtools package and Methods for more information.

3.26. Supplementary Table 26. Study 2: Predictors of Facebook reactions on Ukrainian social media before the invasion.

	Share Pro-Ukrainian Facebook Before	Like Pro-Ukrainian Facebook Before	Comment Pro-Ukrainian Facebook Before	Love Pro-Ukrainian Facebook Before	Wow Pro-Ukrainian Facebook Before	Haha Pro-Ukrainian Facebook Before	Sad Pro-Ukrainian Facebook Before	Angry Pro-Ukrainian Facebook Before
(Intercept)	3.730 (t(99.229)=14.221, p<0.001, d=2.855, 95%CI=[3.111, 4.472])	44.317 (t(78.995)=9.460, p<0.001, d=2.129, 95%CI=[20.204, 97.209])	7.846 (t(81.667)=17.014, p<0.001, d=3.765, 95%CI=[6.189, 9.948])	1.918 (t(74.705)=9.602, p<0.001, d=2.222, 95%CI=[1.679, 2.191])	1.734 (t(58.413)=7.163, p<0.001, d=1.874, 95%CI=[1.491, 2.016])	3.640 (t(82.429)=14.976, p<0.001, d=3.299, 95%CI=[3.074, 4.311])	1.273 (t(61.346)=1.666, p=0.101, d=0.426, 95%CI=[0.958, 1.691])	2.293 (t(241.894)=14.527, p<0.001, d=1.868, 95%CI=[2.050, 2.564])
Ingroup mentions (binary)	1.055 (t(465963.610)=17.453, p<0.001, d=0.051, 95%CI=[1.049, 1.062])	1.123 (t(465899.366)=29.144, p<0.001, d=0.085, 95%CI=[1.115, 1.132])	1.240 (t(465959.274)=57.205, p<0.001, d=0.168, 95%CI=[1.231, 1.249])	1.033 (t(465967.787)=12.105, p<0.001, d=0.035, 95%CI=[1.028, 1.039])	0.993 (t(465958.973)=-2.849, p=0.004, d=0.008, 95%CI=[0.988, 0.998])	1.160 (t(465968.534)=42.048, p<0.001, d=0.123, 95%CI=[1.152, 1.168])	0.992 (t(465929.895)=-2.577, p=0.010, d=0.008, 95%CI=[0.986, 0.998])	1.154 (t(465963.184)=44.653, p<0.001, d=0.131, 95%CI=[1.147, 1.162])
Outgroup mentions (binary)	1.041 (t(458850.293)=9.041, p<0.001, d=0.027, 95%CI=[1.032, 1.050])	1.412 (t(465917.999)=59.322, p<0.001, d=0.174, 95%CI=[1.396, 1.428])	1.275 (t(458412.854)=44.439, p<0.001, d=0.131, 95%CI=[1.261, 1.288])	1.120 (t(454004.118)=28.800, p<0.001, d=0.085, 95%CI=[1.111, 1.128])	0.991 (t(454069.045)=-2.559, p=0.010, d=0.008, 95%CI=[0.983, 0.998])	1.339 (t(454874.095)=56.939, p<0.001, d=0.169, 95%CI=[1.325, 1.352])	0.817 (t(462069.897)=-43.710, p<0.001, d=0.129, 95%CI=[0.810, 0.824])	1.053 (t(462155.294)=11.106, p<0.001, d=0.033, 95%CI=[1.044, 1.063])
Ingroup solidarity	1.156 (t(465949.467)=33.602, p<0.001, d=0.098, 95%CI=[1.146, 1.166])	1.842 (t(465895.254)=109.226, p<0.001, d=0.320, 95%CI=[1.822, 1.862])	1.058 (t(465942.705)=10.746, p<0.001, d=0.031, 95%CI=[1.047, 1.069])	1.604 (t(465947.327)=124.781, p<0.001, d=0.366, 95%CI=[1.592, 1.616])	0.874 (t(465934.982)=-37.919, p<0.001, d=0.111, 95%CI=[0.867, 0.880])	0.881 (t(465949.803)=-25.594, p<0.001, d=0.075, 95%CI=[0.873, 0.890])	1.026 (t(465912.969)=5.744, p<0.001, d=0.017, 95%CI=[1.017, 1.035])	0.839 (t(465964.926)=-39.095, p<0.001, d=0.115, 95%CI=[0.831, 0.846])
Outgroup hostility	1.062 (t(465873.621)=10.766, p<0.001, d=0.032, 95%CI=[1.050, 1.073])	1.005 (t(465879.596)=0.693, p=0.488, d=0.002, 95%CI=[0.991, 1.019])	1.049 (t(465857.241)=7.084, p<0.001, d=0.021, 95%CI=[1.035, 1.063])	0.965 (t(465824.672)=-7.405, p<0.001, d=0.022, 95%CI=[0.955, 0.974])	0.954 (t(465806.601)=-10.297, p<0.001, d=0.030, 95%CI=[0.945, 0.962])	1.092 (t(465835.437)=13.887, p<0.001, d=0.041, 95%CI=[1.079, 1.106])	1.026 (t(465838.586)=4.397, p<0.001, d=0.013, 95%CI=[1.014, 1.037])	1.201 (t(465898.996)=31.659, p<0.001, d=0.093, 95%CI=[1.188, 1.215])
Moral emotional	1.025 (t(465878.499)=13.241, p<0.001, d=0.039, 95%CI=[1.022, 1.029])	1.031 (t(465879.912)=12.437, p<0.001, d=0.036, 95%CI=[1.026, 1.036])	1.022 (t(465862.044)=9.291, p<0.001, d=0.027, 95%CI=[1.017, 1.026])	1.007 (t(465836.469)=4.164, p<0.001, d=0.012, 95%CI=[1.004, 1.010])	1.007 (t(465814.804)=4.476, p<0.001, d=0.013, 95%CI=[1.004, 1.010])	0.974 (t(465847.122)=-11.980, p<0.001, d=0.035, 95%CI=[0.970, 0.978])	1.052 (t(465841.074)=26.061, p<0.001, d=0.076, 95%CI=[1.048, 1.056])	1.047 (t(465915.610)=23.401, p<0.001, d=0.069, 95%CI=[1.043, 1.051])
Positive	0.990 (t(465931.820)=-7.191, p<0.001, d=0.021, 95%CI=[0.988, 0.993])	1.022 (t(465889.931)=12.455, p<0.001, d=0.036, 95%CI=[1.018, 1.025])	0.972 (t(465921.747)=-17.543, p<0.001, d=0.051, 95%CI=[0.969, 0.975])	1.025 (t(465923.606)=21.266, p<0.001, d=0.062, 95%CI=[1.023, 1.028])	0.960 (t(465905.033)=-36.924, p<0.001, d=0.108, 95%CI=[0.958, 0.962])	0.989 (t(465928.411)=-7.501, p<0.001, d=0.022, 95%CI=[0.986, 0.992])	0.941 (t(465891.146)=-43.933, p<0.001, d=0.129, 95%CI=[0.939, 0.944])	0.938 (t(465960.848)=-46.241, p<0.001, d=0.135, 95%CI=[0.935, 0.940])
Negative	1.017 (t(465937.830)=12.513, p<0.001, d=0.037, 95%CI=[1.015, 1.020])	1.008 (t(465891.046)=4.571, p<0.001, d=0.013, 95%CI=[1.005, 1.012])	1.010 (t(465928.397)=6.091, p<0.001, d=0.018, 95%CI=[1.007, 1.014])	0.984 (t(465933.988)=-13.655, p<0.001, d=0.040, 95%CI=[0.981, 0.986])	1.019 (t(465915.197)=16.600, p<0.001, d=0.049, 95%CI=[1.017, 1.021])	0.972 (t(465938.197)=-18.218, p<0.001, d=0.053, 95%CI=[0.969, 0.975])	1.085 (t(465896.634)=57.287, p<0.001, d=0.168, 95%CI=[1.082, 1.088])	1.035 (t(465966.408)=23.927, p<0.001, d=0.070, 95%CI=[1.032, 1.038])
Total tokens	1.061 (t(465947.365)=22.593, p<0.001, d=0.066, 95%CI=[1.056, 1.067])	1.003 (t(465887.872)=0.974, p=0.330, d=0.003, 95%CI=[0.997, 1.010])	1.016 (t(465935.237)=5.074, p<0.001, d=0.015, 95%CI=[1.010, 1.023])	0.957 (t(465962.807)=-18.966, p<0.001, d=0.056, 95%CI=[0.953, 0.961])	1.029 (t(465931.058)=13.231, p<0.001, d=0.039, 95%CI=[1.025, 1.034])	1.000 (t(465965.915)=-0.100, p=0.920, d=0.000, 95%CI=[0.994, 1.006])	1.005 (t(465890.382)=1.947, p=0.051, d=0.006, 95%CI=[1.000, 1.011])	1.042 (t(465879.433)=14.810, p<0.001, d=0.043, 95%CI=[1.036, 1.047])
Followers at posting	3.304 (t(406.782)=15.206, p<0.001, d=1.508, 95%CI=[2.833, 3.855])	552.498 (t(31233.531)=48.907, p<0.001, d=0.553, 95%CI=[428.975, 711.590])	7.195 (t(430.254)=19.921, p<0.001, d=1.921, 95%CI=[5.926, 8.737])	2.581 (t(160.029)=15.263, p<0.001, d=2.413, 95%CI=[2.285, 2.915])	0.717 (t(242.312)=-5.115, p<0.001, d=0.657, 95%CI=[0.631, 0.814])	3.429 (t(161.592)=15.482, p<0.001, d=2.436, 95%CI=[2.934, 4.008])	0.262 (t(1170.312)=-14.288, p<0.001, d=0.835, 95%CI=[0.218, 0.315])	1.872 (t(136.164)=11.826, p<0.001, d=2.027, 95%CI=[1.688, 2.078])
Has media	1.602 (t(465900.788)=13.143, p<0.001, d=0.039, 95%CI=[1.493, 1.719])	1.484 (t(465883.755)=8.484, p<0.001, d=0.025, 95%CI=[1.355, 1.625])	0.782 (t(465886.870)=-5.632, p<0.001, d=0.017, 95%CI=[0.717, 0.852])	1.690 (t(465873.152)=16.655, p<0.001, d=0.049, 95%CI=[1.589, 1.797])	1.014 (t(465852.504)=0.472, p=0.637, d=0.001, 95%CI=[0.957, 1.075])	0.738 (t(465881.334)=-7.402, p<0.001, d=0.022, 95%CI=[0.681, 0.800])	1.076 (t(465861.188)=1.976, p=0.048, d=0.006, 95%CI=[1.001, 1.157])	0.910 (t(465934.531)=-2.530, p=0.011, d=0.007, 95%CI=[0.845, 0.979])
Has URL	0.669 (t(465892.181)=-11.303, p<0.001, d=0.033, 95%CI=[0.624, 0.718])	0.634 (t(465882.853)=-9.893, p<0.001, d=0.029, 95%CI=[0.579, 0.694])	0.529 (t(465878.010)=-14.713, p<0.001, d=0.043, 95%CI=[0.486, 0.576])	0.827 (t(465854.560)=-6.085, p<0.001, d=0.018, 95%CI=[0.778, 0.879])	0.860 (t(465838.003)=-5.152, p<0.001, d=0.015, 95%CI=[0.811, 0.911])	0.695 (t(465863.179)=-8.952, p<0.001, d=0.026, 95%CI=[0.642, 0.753])	0.871 (t(465855.590)=-3.780, p<0.001, d=0.011, 95%CI=[0.810, 0.935])	0.803 (t(465912.469)=-5.910, p<0.001, d=0.017, 95%CI=[0.747, 0.864])
N	465982	465982	465982	465982	465982	465982	465982	465982
N (Page.Name)	84	84	84	84	84	84	84	84
AIC	1282699.170	1525480.564	1468326.092	1162009.392	1106026.790	1409030.008	1313059.768	1322954.620
BIC	1282853.896	1525635.290	1468480.819	1162164.119	1106181.517	1409184.734	1313214.495	1323109.346
R2 (fixed)	0.467	0.700	0.587	0.464	0.093	0.445	0.378	0.241
R2 (total)	0.672	0.969	0.763	0.616	0.445	0.595	0.763	0.335

Mixed-effects linear regression. DV is log-transformed engagement. Estimates are exponentiated. P-values estimated using Satterthwaite d.f. Cohen's d (d) estimated using effectsize::t_to_d(t,df_error). See the jtools package and Methods for more information.

3.27. Supplementary Table 27. Study 2: Predictors of Facebook reactions on Ukrainian social media after the invasion.

	Share Pro-Ukrainian Facebook After	Like Pro-Ukrainian Facebook After	Comment Pro-Ukrainian Facebook After	Love Pro-Ukrainian Facebook After	Wow Pro-Ukrainian Facebook After	Haha Pro-Ukrainian Facebook After	Sad Pro-Ukrainian Facebook After	Angry Pro-Ukrainian Facebook After
(Intercept)	3.764 (t(83.986)=6.055, p<0.001, d=1.322, 95%CI=[2.451, 5.781])	19.416 (t(84.489)=12.623, p<0.001, d=2.747, 95%CI=[12.251, 30.773])	3.825 (t(85.649)=10.045, p<0.001, d=2.171, 95%CI=[2.944, 4.970])	2.417 (t(87.542)=8.031, p<0.001, d=1.717, 95%CI=[1.948, 2.997])	1.795 (t(94.603)=13.804, p<0.001, d=2.838, 95%CI=[1.652, 1.950])	1.697 (t(120.773)=13.697, p<0.001, d=2.493, 95%CI=[1.573, 1.830])	2.736 (t(89.260)=10.346, p<0.001, d=2.190, 95%CI=[2.261, 3.311])	1.821 (t(89.448)=6.806, p<0.001, d=1.439, 95%CI=[1.532, 2.164])
Ingroup mentions (binary)	0.992 (t(533141.205)=-2.454, p=0.014, d=0.007, 95%CI=[0.985, 0.998])	0.961 (t(533143.680)=-9.131, p<0.001, d=0.025, 95%CI=[0.952, 0.969])	1.094 (t(533152.093)=23.819, p<0.001, d=0.065, 95%CI=[1.086, 1.102])	0.881 (t(533162.194)=-31.239, p<0.001, d=0.086, 95%CI=[0.874, 0.888])	0.986 (t(533186.510)=-6.166, p<0.001, d=0.017, 95%CI=[0.982, 0.991])	0.931 (t(533218.989)=-22.065, p<0.001, d=0.060, 95%CI=[0.925, 0.936])	0.947 (t(533169.645)=-13.295, p<0.001, d=0.036, 95%CI=[0.939, 0.954])	1.123 (t(533169.721)=31.158, p<0.001, d=0.085, 95%CI=[1.115, 1.131])
Outgroup mentions (binary)	1.054 (t(533143.164)=15.678, p<0.001, d=0.043, 95%CI=[1.047, 1.061])	1.172 (t(533146.636)=35.838, p<0.001, d=0.098, 95%CI=[1.162, 1.183])	1.164 (t(533158.572)=39.775, p<0.001, d=0.109, 95%CI=[1.155, 1.172])	1.084 (t(533172.215)=19.621, p<0.001, d=0.054, 95%CI=[1.075, 1.092])	1.024 (t(533201.086)=10.564, p<0.001, d=0.029, 95%CI=[1.020, 1.029])	1.252 (t(533203.855)=68.151, p<0.001, d=0.187, 95%CI=[1.244, 1.260])	0.821 (t(533181.724)=-47.333, p<0.001, d=0.130, 95%CI=[0.815, 0.828])	1.137 (t(533181.761)=34.108, p<0.001, d=0.093, 95%CI=[1.128, 1.145])
Ingroup solidarity	1.237 (t(533140.210)=58.969, p<0.001, d=0.162, 95%CI=[1.228, 1.246])	2.448 (t(533142.277)=188.538, p<0.001, d=0.516, 95%CI=[2.425, 2.471])	1.128 (t(533149.381)=29.464, p<0.001, d=0.081, 95%CI=[1.119, 1.137])	2.361 (t(533158.171)=196.399, p<0.001, d=0.538, 95%CI=[2.340, 2.381])	0.879 (t(533180.333)=-52.838, p<0.001, d=0.145, 95%CI=[0.874, 0.883])	0.891 (t(533217.734)=-32.789, p<0.001, d=0.090, 95%CI=[0.885, 0.897])	0.879 (t(533164.800)=-28.990, p<0.001, d=0.079, 95%CI=[0.871, 0.887])	0.608 (t(533164.889)=-123.603, p<0.001, d=0.339, 95%CI=[0.604, 0.613])
Outgroup hostility	1.096 (t(533138.926)=25.532, p<0.001, d=0.070, 95%CI=[1.088, 1.103])	0.841 (t(533140.385)=-36.774, p<0.001, d=0.101, 95%CI=[0.833, 0.848])	0.975 (t(533145.310)=-6.136, p<0.001, d=0.017, 95%CI=[0.968, 0.983])	0.858 (t(533151.640)=-35.274, p<0.001, d=0.097, 95%CI=[0.851, 0.865])	0.959 (t(533168.750)=-17.385, p<0.001, d=0.048, 95%CI=[0.954, 0.963])	1.021 (t(533207.026)=5.965, p<0.001, d=0.016, 95%CI=[1.014, 1.028])	1.245 (t(533156.566)=49.654, p<0.001, d=0.136, 95%CI=[1.235, 1.256])	1.498 (t(533156.683)=101.188, p<0.001, d=0.277, 95%CI=[1.487, 1.510])
Moral emotional	1.021 (t(533137.639)=11.919, p<0.001, d=0.033, 95%CI=[1.017, 1.024])	0.993 (t(533138.416)=-2.875, p=0.004, d=0.008, 95%CI=[0.989, 0.998])	0.999 (t(533140.653)=-0.288, p=0.773, d=0.001, 95%CI=[0.996, 1.003])	0.999 (t(533143.602)=-0.283, p=0.777, d=0.001, 95%CI=[0.995, 1.004])	0.987 (t(533152.243)=-10.723, p<0.001, d=0.029, 95%CI=[0.985, 0.990])	0.969 (t(533175.276)=-18.398, p<0.001, d=0.050, 95%CI=[0.966, 0.973])	1.080 (t(533145.967)=36.196, p<0.001, d=0.099, 95%CI=[1.076, 1.085])	1.026 (t(533146.122)=13.481, p<0.001, d=0.037, 95%CI=[1.023, 1.030])
Positive	0.987 (t(533139.070)=-9.252, p<0.001, d=0.025, 95%CI=[0.984, 0.989])	1.016 (t(533140.565)=8.271, p<0.001, d=0.023, 95%CI=[1.012, 1.020])	1.001 (t(533145.528)=0.525, p=0.600, d=0.001, 95%CI=[0.998, 1.004])	1.001 (t(533151.796)=0.528, p=0.597, d=0.001, 95%CI=[0.997, 1.004])	0.981 (t(533168.395)=-19.163, p<0.001, d=0.052, 95%CI=[0.979, 0.983])	1.003 (t(533204.288)=1.866, p=0.062, d=0.005, 95%CI=[1.000, 1.005])	0.939 (t(533156.621)=-35.089, p<0.001, d=0.096, 95%CI=[0.936, 0.943])	0.947 (t(533156.738)=-33.634, p<0.001, d=0.092, 95%CI=[0.944, 0.950])
Negative	0.995 (t(533144.417)=-3.790, p<0.001, d=0.010, 95%CI=[0.993, 0.998])	1.014 (t(533148.492)=8.397, p<0.001, d=0.023, 95%CI=[1.011, 1.017])	0.990 (t(533162.657)=-7.126, p<0.001, d=0.020, 95%CI=[0.987, 0.993])	1.003 (t(533178.731)=1.669, p=0.095, d=0.005, 95%CI=[1.000, 1.006])	0.994 (t(533210.056)=-6.877, p<0.001, d=0.019, 95%CI=[0.992, 0.996])	0.974 (t(533148.787)=-21.229, p<0.001, d=0.058, 95%CI=[0.972, 0.976])	1.002 (t(533189.677)=1.279, p=0.201, d=0.004, 95%CI=[0.999, 1.005])	0.991 (t(533189.691)=-6.585, p<0.001, d=0.018, 95%CI=[0.988, 0.993])
Total tokens	1.019 (t(533173.502)=7.882, p<0.001, d=0.022, 95%CI=[1.014, 1.024])	0.953 (t(533188.354)=-15.310, p<0.001, d=0.042, 95%CI=[0.947, 0.959])	1.023 (t(533218.251)=8.399, p<0.001, d=0.023, 95%CI=[1.018, 1.029])	0.914 (t(533192.387)=-30.928, p<0.001, d=0.085, 95%CI=[0.909, 0.919])	1.045 (t(532724.225)=26.893, p<0.001, d=0.074, 95%CI=[1.041, 1.048])	1.037 (t(526931.718)=15.613, p<0.001, d=0.043, 95%CI=[1.033, 1.042])	1.048 (t(533119.987)=15.755, p<0.001, d=0.043, 95%CI=[1.042, 1.054])	1.031 (t(533120.727)=11.414, p<0.001, d=0.031, 95%CI=[1.026, 1.036])
Followers at posting	0.244 (t(523767.287)=-114.748, p<0.001, d=0.317, 95%CI=[0.238, 0.250])	0.407 (t(510765.913)=-55.495, p<0.001, d=0.155, 95%CI=[0.395, 0.420])	0.825 (t(425512.246)=-13.838, p<0.001, d=0.042, 95%CI=[0.803, 0.848])	0.647 (t(300744.837)=-29.342, p<0.001, d=0.107, 95%CI=[0.628, 0.666])	1.100 (t(112753.596)=11.533, p<0.001, d=0.069, 95%CI=[1.082, 1.118])	1.361 (t(18132.930)=26.498, p<0.001, d=0.394, 95%CI=[1.331, 1.393])	0.745 (t(223637.295)=-19.514, p<0.001, d=0.083, 95%CI=[0.724, 0.768])	0.776 (t(224297.194)=-18.651, p<0.001, d=0.079, 95%CI=[0.755, 0.797])
Has media	1.164 (t(533143.697)=9.268, p<0.001, d=0.025, 95%CI=[1.127, 1.202])	0.818 (t(533147.101)=-9.293, p<0.001, d=0.025, 95%CI=[0.784, 0.853])	1.007 (t(533157.566)=0.361, p=0.718, d=0.001, 95%CI=[0.971, 1.044])	1.188 (t(533168.009)=8.634, p<0.001, d=0.024, 95%CI=[1.142, 1.235])	0.932 (t(533189.140)=-6.299, p<0.001, d=0.017, 95%CI=[0.912, 0.953])	1.090 (t(533218.649)=5.378, p<0.001, d=0.015, 95%CI=[1.056, 1.125])	1.008 (t(533174.876)=0.378, p=0.705, d=0.001, 95%CI=[0.968, 1.048])	0.916 (t(533174.939)=-4.811, p<0.001, d=0.013, 95%CI=[0.883, 0.949])
Has URL	0.418 (t(533143.491)=-54.098, p<0.001, d=0.148, 95%CI=[0.405, 0.431])	0.402 (t(533146.881)=-42.885, p<0.001, d=0.117, 95%CI=[0.385, 0.419])	0.659 (t(533157.614)=-22.863, p<0.001, d=0.063, 95%CI=[0.636, 0.683])	0.588 (t(533168.652)=-27.136, p<0.001, d=0.074, 95%CI=[0.566, 0.611])	0.872 (t(533191.537)=-12.529, p<0.001, d=0.034, 95%CI=[0.853, 0.891])	1.020 (t(533218.845)=1.255, p=0.209, d=0.003, 95%CI=[0.989, 1.052])	0.624 (t(533176.055)=-23.718, p<0.001, d=0.065, 95%CI=[0.600, 0.648])	0.872 (t(533176.117)=-7.616, p<0.001, d=0.021, 95%CI=[0.842, 0.903])
N	533231	533231	533231	533231	533231	533231	533231	533231
N (Page.Name)	84	84	84	84	84	84	84	84
AIC	1618598.393	1912856.834	1750087.864	1824867.388	1206288.855	1596279.977	1842648.310	1735377.336
BIC	1618755.007	1913013.447	1750244.478	1825024.002	1206445.469	1596436.591	1842804.924	1735533.950
R2 (fixed)	0.316	0.146	0.026	0.134	0.021	0.090	0.059	0.079
R2 (total)	0.840	0.730	0.496	0.438	0.213	0.161	0.329	0.344

Mixed-effects linear regression. DV is log-transformed engagement. Estimates are exponentiated. P-values estimated using Satterthwaite d.f. Cohen’s d (d) estimated using effectsize::t_to_d(t,df_error). See the jtools package and Methods for more information.

3.28. Supplementary Table 28. Study 2 Variance Inflation Factors.

Term	VIF	VIF_CI_low	VIF_CI_high	Model
ingroup mentions (binary)	1.07349925128342	1.06839233882132	1.07898750111963	Pro-Ukrainian Twitter Before
outgroup mentions (binary)	1.23522994786479	1.22854121800226	1.24211443719496	Pro-Ukrainian Twitter Before
ingroup solidarity	1.09761879049528	1.09225610527419	1.10329319918111	Pro-Ukrainian Twitter Before
outgroup hostility	1.24634982020103	1.23955693497881	1.25333532472541	Pro-Ukrainian Twitter Before
moral-emotional	1.20650560676194	1.20008691498267	1.2131302070793	Pro-Ukrainian Twitter Before
positive	1.11380246215016	1.10827617390289	1.11961080563352	Pro-Ukrainian Twitter Before
negative	1.22596780148746	1.2193659755568	1.23276830957703	Pro-Ukrainian Twitter Before
total tokens	1.11515511881123	1.10961534381597	1.12097486471138	Pro-Ukrainian Twitter Before
follower count	1.00218805929293	1.00026309694242	1.01819710797598	Pro-Ukrainian Twitter Before
ingroup mentions (binary)	1.06860069397186	1.06395735834215	1.07358113804897	Pro-Ukrainian Twitter After
outgroup mentions (binary)	1.13197043128647	1.1267333847689	1.13742389004836	Pro-Ukrainian Twitter After
ingroup solidarity	1.11178785305927	1.10673259157561	1.11708255095398	Pro-Ukrainian Twitter After
outgroup hostility	1.14777757375461	1.14240038977754	1.15335780568379	Pro-Ukrainian Twitter After
moral-emotional	1.40514894636328	1.39756706205556	1.41287542255282	Pro-Ukrainian Twitter After
positive	1.11616929579622	1.1110742249927	1.12149808190584	Pro-Ukrainian Twitter After
negative	1.45908592563966	1.45104733267474	1.46726778289676	Pro-Ukrainian Twitter After
total tokens	1.13388463947136	1.12863053510284	1.13935335550028	Pro-Ukrainian Twitter After
follower count	1.0017074217246	1.00014232385946	1.02048348714479	Pro-Ukrainian Twitter After
ingroup mentions (binary)	1.08180821060391	1.07853463633924	1.08521823789065	Pro-Ukrainian Facebook Before
outgroup mentions (binary)	1.38057018694667	1.37554719992052	1.38566035700246	Pro-Ukrainian Facebook Before
ingroup solidarity	1.12609369472978	1.12254929442813	1.12974060703328	Pro-Ukrainian Facebook Before
outgroup hostility	1.39427743443479	1.38917559038443	1.39944616041031	Pro-Ukrainian Facebook Before
moral-emotional	1.34614439205224	1.34131958442536	1.35103740194381	Pro-Ukrainian Facebook Before
positive	1.36694666546369	1.36200208129494	1.37195878767674	Pro-Ukrainian Facebook Before
negative	1.39634202512211	1.39122830582496	1.40152258550581	Pro-Ukrainian Facebook Before
total tokens	1.53231041897336	1.52641554633246	1.53827130319712	Pro-Ukrainian Facebook Before
followers at posting	1.01007173192865	1.00753021940314	1.01347102635552	Pro-Ukrainian Facebook Before
has media	39.7798830595034	39.5549839085451	40.0060940934656	Pro-Ukrainian Facebook Before
has URL	39.7205591815138	39.4959997054359	39.9464285848228	Pro-Ukrainian Facebook Before
ingroup mentions (binary)	1.07720619402243	1.07416912806872	1.0803676212818	Pro-Ukrainian Facebook After
outgroup mentions (binary)	1.18324968164488	1.17961848996301	1.18695428198882	Pro-Ukrainian Facebook After
ingroup solidarity	1.12960538467793	1.12626937870372	1.13302952711066	Pro-Ukrainian Facebook After
outgroup hostility	1.2215491497239	1.21770956948597	1.22545644575604	Pro-Ukrainian Facebook After
moral-emotional	1.76235144661145	1.75560523192641	1.76915789302948	Pro-Ukrainian Facebook After
positive	1.50481024484144	1.49944518540353	1.51023293595468	Pro-Ukrainian Facebook After
negative	1.95900380508227	1.95120383665115	1.96686773404969	Pro-Ukrainian Facebook After
total tokens	1.86047832541474	1.85320624691539	1.86781238555791	Pro-Ukrainian Facebook After
followers at posting	1.01040466812366	1.00799589118814	1.01353909354394	Pro-Ukrainian Facebook After
has media	9.5832628989777	9.53464261257883	9.63216016618955	Pro-Ukrainian Facebook After
has URL	9.64756800130311	9.59860344994867	9.69681138017916	Pro-Ukrainian Facebook After

3.29. Supplementary Table 29. Study 3 Variance Inflation Factors.

Term	VIF	VIF_CI_low	VIF_CI_high	Model
ingroup mentions (binary)	1.0894954990868	1.08346480140857	1.09596194110123	Pro-Ukrainian Geolocated Twitter Before
outgroup mentions (binary)	1.08729509595422	1.08129133537695	1.09374226345675	Pro-Ukrainian Geolocated Twitter Before
ingroup solidarity	1.09016702906546	1.0841281463783	1.09663939454855	Pro-Ukrainian Geolocated Twitter Before
outgroup hostility	1.08148173460941	1.07555009527335	1.08787908275876	Pro-Ukrainian Geolocated Twitter Before
positive	1.21787721688211	1.2104051222367	1.22561466722703	Pro-Ukrainian Geolocated Twitter Before
negative	1.20436188317146	1.19703631917296	1.21195980248049	Pro-Ukrainian Geolocated Twitter Before
moral-emotional	1.18701454148342	1.17987792301831	1.19443430377331	Pro-Ukrainian Geolocated Twitter Before
total tokens	1.34747603840714	1.33861513167886	1.35656881802208	Pro-Ukrainian Geolocated Twitter Before
follower count	1.06421351163641	1.05850739973646	1.0704761294341	Pro-Ukrainian Geolocated Twitter Before
verified	1.06416365489268	1.05845822718396	1.07042592304812	Pro-Ukrainian Geolocated Twitter Before
has URL	1.05251608784578	1.04697849682508	1.05870642249143	Pro-Ukrainian Geolocated Twitter Before
ingroup mentions (binary)	1.12616175967724	1.11996588885857	1.13267762825167	Pro-Ukrainian Geolocated Twitter After
outgroup mentions (binary)	1.10496473329687	1.09900264319167	1.11128586955758	Pro-Ukrainian Geolocated Twitter After
ingroup solidarity	1.14770619907566	1.14127847220006	1.15442636734127	Pro-Ukrainian Geolocated Twitter After
outgroup hostility	1.10780768185608	1.10181379203951	1.1141544385526	Pro-Ukrainian Geolocated Twitter After
positive	1.23298146500534	1.22566190829324	1.24053843843906	Pro-Ukrainian Geolocated Twitter After
negative	1.39026549270622	1.38133638245098	1.39940368086123	Pro-Ukrainian Geolocated Twitter After
moral-emotional	1.34131497574002	1.33288465571776	1.34995879402499	Pro-Ukrainian Geolocated Twitter After
total tokens	1.41415317606426	1.40498105432373	1.42353303052791	Pro-Ukrainian Geolocated Twitter After
follower count	1.02780028236294	1.02292929979121	1.03370603143126	Pro-Ukrainian Geolocated Twitter After
verified	1.02847843859596	1.02359092579333	1.03437853486416	Pro-Ukrainian Geolocated Twitter After
has URL	1.05400468661353	1.04866626754426	1.05992870058452	Pro-Ukrainian Geolocated Twitter After

3.30. Supplementary Table 30. Study 3: Predictors of engagement on geolocated Ukrainian Twitter before and after the invasion for BERT-NLI classification without the .999 probability threshold do not change.

	Pro-Ukrainian Geolocated Twitter Before (not .999)	Pro-Ukrainian Geolocated Twitter After (not .999)	Pro-Ukrainian Geolocated Twitter Before Full (not .999)	Pro-Ukrainian Geolocated Twitter After Full (not .999)
(Intercept)	2.149 (t(7349.727)=35.271, p<0.001, d=0.823, 95%CI=[2.059, 2.242])	2.190 (t(9743.077)=48.211, p<0.001, d=0.977, 95%CI=[2.121, 2.261])	1.919 (t(5336.831)=42.123, p<0.001, d=1.153, 95%CI=[1.862, 1.978])	2.254 (t(3618.914)=38.786, p<0.001, d=1.289, 95%CI=[2.163, 2.348])
Ingroup mentions (binary)	1.070 (t(135306.087)=9.579, p<0.001, d=0.052, 95%CI=[1.055, 1.085])	1.062 (t(147055.819)=9.557, p<0.001, d=0.050, 95%CI=[1.049, 1.075])	1.070 (t(93706.977)=7.932, p<0.001, d=0.052, 95%CI=[1.052, 1.087])	1.072 (t(76463.139)=7.412, p<0.001, d=0.054, 95%CI=[1.052, 1.091])
Outgroup mentions (binary)	1.056 (t(133477.245)=3.558, p<0.001, d=0.019, 95%CI=[1.025, 1.089])	1.035 (t(146094.978)=3.789, p<0.001, d=0.020, 95%CI=[1.017, 1.054])	1.072 (t(92518.893)=3.737, p<0.001, d=0.025, 95%CI=[1.033, 1.111])	1.051 (t(75683.386)=3.997, p<0.001, d=0.029, 95%CI=[1.026, 1.077])
Ingroup solidarity	1.158 (t(134160.700)=16.698, p<0.001, d=0.091, 95%CI=[1.138, 1.178])	1.141 (t(146948.664)=18.751, p<0.001, d=0.098, 95%CI=[1.126, 1.157])	1.170 (t(92898.959)=14.709, p<0.001, d=0.097, 95%CI=[1.146, 1.195])	1.161 (t(76130.412)=14.748, p<0.001, d=0.107, 95%CI=[1.139, 1.185])
Outgroup hostility	1.135 (t(133023.064)=9.500, p<0.001, d=0.052, 95%CI=[1.106, 1.166])	1.066 (t(146111.701)=8.751, p<0.001, d=0.046, 95%CI=[1.051, 1.082])	1.093 (t(92077.901)=5.363, p<0.001, d=0.035, 95%CI=[1.058, 1.129])	1.057 (t(75681.221)=5.373, p<0.001, d=0.039, 95%CI=[1.036, 1.079])
Positive	1.015 (t(133609.283)=5.805, p<0.001, d=0.032, 95%CI=[1.010, 1.020])	1.003 (t(146344.832)=1.006, p=0.314, d=0.005, 95%CI=[0.997, 1.009])	1.014 (t(92544.765)=4.397, p<0.001, d=0.029, 95%CI=[1.008, 1.021])	0.999 (t(75928.477)=-0.328, p=0.743, d=0.002, 95%CI=[0.990, 1.007])
Negative	0.999 (t(132819.101)=-0.147, p=0.883, d=0.001, 95%CI=[0.993, 1.006])	0.986 (t(145571.483)=-4.199, p<0.001, d=0.022, 95%CI=[0.980, 0.993])	0.996 (t(91977.819)=-0.917, p=0.359, d=0.006, 95%CI=[0.987, 1.005])	0.988 (t(75605.633)=-2.431, p=0.015, d=0.018, 95%CI=[0.979, 0.998])
Moral emotional	1.016 (t(133125.772)=3.420, p=0.001, d=0.019, 95%CI=[1.007, 1.026])	1.016 (t(146103.857)=3.531, p<0.001, d=0.018, 95%CI=[1.007, 1.025])	1.022 (t(92152.299)=3.674, p<0.001, d=0.024, 95%CI=[1.010, 1.034])	1.012 (t(75777.064)=1.817, p=0.069, d=0.013, 95%CI=[0.999, 1.025])
Total tokens	1.068 (t(133934.982)=25.056, p<0.001, d=0.137, 95%CI=[1.063, 1.073])	1.083 (t(146533.046)=27.914, p<0.001, d=0.146, 95%CI=[1.077, 1.089])	1.071 (t(92716.714)=21.076, p<0.001, d=0.138, 95%CI=[1.064, 1.077])	1.105 (t(75996.608)=23.484, p<0.001, d=0.170, 95%CI=[1.096, 1.114])
Followers count	1.179 (t(6154.656)=9.499, p<0.001, d=0.242, 95%CI=[1.140, 1.220])	1.029 (t(14855.954)=7.362, p<0.001, d=0.121, 95%CI=[1.021, 1.037])	1.179 (t(4164.768)=9.610, p<0.001, d=0.298, 95%CI=[1.140, 1.219])	1.030 (t(6277.078)=7.198, p<0.001, d=0.182, 95%CI=[1.021, 1.038])
User is not verified	0.894 (t(6884.610)=-4.303, p<0.001, d=0.104, 95%CI=[0.849, 0.941])	1.005 (t(8019.980)=0.203, p=0.839, d=0.005, 95%CI=[0.957, 1.056])		
User is verified	2.998 (t(5961.185)=9.994, p<0.001, d=0.259, 95%CI=[2.417, 3.718])	7.242 (t(7126.757)=16.376, p<0.001, d=0.388, 95%CI=[5.714, 9.178])	3.337 (t(3995.520)=11.174, p<0.001, d=0.354, 95%CI=[2.701, 4.122])	7.080 (t(2759.779)=15.843, p<0.001, d=0.603, 95%CI=[5.557, 9.019])
Has URL	1.136 (t(136089.760)=23.489, p<0.001, d=0.127, 95%CI=[1.124, 1.148])	1.001 (t(147555.445)=0.098, p=0.922, d=0.001, 95%CI=[0.990, 1.012])	1.138 (t(94301.344)=19.418, p<0.001, d=0.126, 95%CI=[1.123, 1.152])	0.969 (t(76550.769)=-3.928, p<0.001, d=0.028, 95%CI=[0.954, 0.984])
N	136950	149230	94795	77251
N (author_id)	6446	7596	4332	2922
AIC	308466.894	371213.037	217671.652	201639.190
BIC	308614.305	371361.735	217804.085	201768.757
R2 (fixed)	0.040	0.051	0.052	0.082
R2 (total)	0.615	0.594	0.600	0.577

Mixed-effects linear regression. DV is log-transformed engagement. Estimates are exponentiated. P-values estimated using Satterthwaite d.f. Cohen’s d (d) estimated using effectsize::t_to_d(t,df_error). See the jtools package and Methods for more information.

3.31. Supplementary Table 31. Study 2: Predictors of engagement on Ukrainian social media after the invasion with the dictionary approach.

	Pro-Ukrainian Twitter Before	Pro-Ukrainian Twitter After	Pro-Ukrainian Facebook Before	Pro-Ukrainian Facebook After
(Intercept)	3.930 (t(62.692)=11.348, p<0.001, d=2.866, 95%CI=[3.103, 4.978])	2.791 (t(55.607)=1.753, p=0.085, d=0.470, 95%CI=[0.886, 8.789])	52.806 (t(76.439)=15.865, p<0.001, d=3.629, 95%CI=[32.350, 86.198])	41.703 (t(84.200)=15.244, p<0.001, d=3.323, 95%CI=[25.815, 67.371])
Ingroup mentions	1.156 (t(180345.244)=46.154, p<0.001, d=0.217, 95%CI=[1.149, 1.163])	1.108 (t(214815.827)=34.166, p<0.001, d=0.147, 95%CI=[1.101, 1.114])	1.109 (t(465917.736)=59.731, p<0.001, d=0.175, 95%CI=[1.105, 1.112])	1.035 (t(533145.580)=21.470, p<0.001, d=0.059, 95%CI=[1.032, 1.038])
Outgroup mentions	1.232 (t(180334.719)=45.090, p<0.001, d=0.212, 95%CI=[1.220, 1.243])	1.064 (t(214808.760)=19.548, p<0.001, d=0.084, 95%CI=[1.057, 1.070])	1.161 (t(464483.496)=64.338, p<0.001, d=0.189, 95%CI=[1.156, 1.167])	1.083 (t(533151.454)=46.942, p<0.001, d=0.129, 95%CI=[1.080, 1.087])
Ingroup solidarity (dictionary)	1.192 (t(180298.591)=21.687, p<0.001, d=0.102, 95%CI=[1.173, 1.211])	1.305 (t(214804.190)=42.792, p<0.001, d=0.185, 95%CI=[1.289, 1.321])	1.132 (t(465896.155)=30.330, p<0.001, d=0.089, 95%CI=[1.123, 1.141])	1.232 (t(533138.831)=69.133, p<0.001, d=0.189, 95%CI=[1.225, 1.240])
Outgroup hostility (dictionary)	1.004 (t(180296.523)=0.485, p=0.628, d=0.002, 95%CI=[0.989, 1.018])	1.046 (t(214802.650)=10.611, p<0.001, d=0.046, 95%CI=[1.037, 1.055])	0.981 (t(465883.448)=-4.619, p<0.001, d=0.014, 95%CI=[0.973, 0.989])	1.026 (t(533139.455)=11.866, p<0.001, d=0.033, 95%CI=[1.022, 1.030])
Moral emotional	1.044 (t(180298.512)=9.814, p<0.001, d=0.046, 95%CI=[1.035, 1.053])	1.050 (t(214803.368)=12.438, p<0.001, d=0.054, 95%CI=[1.042, 1.058])	1.059 (t(465871.908)=22.353, p<0.001, d=0.065, 95%CI=[1.054, 1.065])	1.036 (t(533137.964)=16.995, p<0.001, d=0.047, 95%CI=[1.032, 1.041])
Positive	1.028 (t(180304.176)=8.346, p<0.001, d=0.039, 95%CI=[1.021, 1.034])	1.054 (t(214802.786)=15.197, p<0.001, d=0.066, 95%CI=[1.047, 1.061])	1.011 (t(465885.665)=6.056, p<0.001, d=0.018, 95%CI=[1.007, 1.014])	1.015 (t(533139.463)=8.191, p<0.001, d=0.022, 95%CI=[1.011, 1.018])
Negative	1.031 (t(180308.203)=9.308, p<0.001, d=0.044, 95%CI=[1.024, 1.038])	0.999 (t(214802.821)=-0.431, p=0.666, d=0.002, 95%CI=[0.993, 1.004])	1.022 (t(465912.913)=11.725, p<0.001, d=0.034, 95%CI=[1.018, 1.026])	0.995 (t(533144.082)=-3.388, p=0.001, d=0.009, 95%CI=[0.992, 0.998])
Total tokens	1.083 (t(180356.857)=23.580, p<0.001, d=0.111, 95%CI=[1.075, 1.090])	1.047 (t(214818.483)=15.106, p<0.001, d=0.065, 95%CI=[1.041, 1.054])	0.925 (t(465915.029)=-20.775, p<0.001, d=0.061, 95%CI=[0.918, 0.932])	0.877 (t(533179.621)=-41.841, p<0.001, d=0.115, 95%CI=[0.871, 0.882])
Followers count	2.138 (t(7022.587)=15.449, p<0.001, d=0.369, 95%CI=[1.941, 2.354])	0.014 (t(12629.495)=-24.676, p<0.001, d=0.439, 95%CI=[0.010, 0.020])		
Followers at posting			62.155 (t(4233.241)=32.056, p<0.001, d=0.985, 95%CI=[48.286, 80.008])	0.454 (t(519713.000)=-52.819, p<0.001, d=0.147, 95%CI=[0.441, 0.468])
Has media			1.428 (t(465882.956)=7.352, p<0.001, d=0.022, 95%CI=[1.299, 1.571])	0.931 (t(533144.946)=-3.576, p<0.001, d=0.010, 95%CI=[0.895, 0.968])
Has URL			0.661 (t(465879.759)=-8.619, p<0.001, d=0.025, 95%CI=[0.602, 0.726])	0.429 (t(533144.748)=-43.148, p<0.001, d=0.118, 95%CI=[0.413, 0.446])
N	180367	214882	465982	533231
N (accounts)	63	63	84	64
AIC	453483.021	598678.699	1564351.611	1827060.993
BIC	453604.254	598802.033	1564506.338	1827217.607
R2 (fixed)	0.256	0.466	0.688	0.108
R2 (total)	0.670	0.977	0.921	0.763

Mixed-effects linear regression. DV is log-transformed engagement. Estimates are exponentiated. P-values estimated using Satterthwaite d.f. Cohen's d (d) estimated using effectsize::t_to_d(t,df_error). See the jtools package and Methods for more information.
