# Supplementary material for: Social identity correlates of social media engagement before and after the 2022 Russian invasion of Ukraine
Source: Nat Commun. 2024 Oct 1;15:8127. doi: 10.1038/s41467-024-52179-8 (PMC11445580; doi:10.1038/s41467-024-52179-8)
Supplement: Supplementary file 2 — Reporting Summary [file 41467_2024_52179_MOESM2_ESM.pdf]

nature portfolio

Corresponding author(s): Yara Kyrychenko , Jon Roozenbeek

Last updated by author(s): Jun 16, 2024

Reporting Summary

Nature Portfolio wishes to improve the reproducibility of the work that we publish. This form provides structure for consistency and transparency in reporting. For further information on Nature Portfolio policies, see our Editorial Policies and the Editorial Policy Checklist.

Statistics

For all statistical analyses, confirm that the following items are present in the figure legend, table legend, main text, or Methods section.

n/a	Confirmed	
☐	☒	The exact sample size (n) for each experimental group/condition, given as a discrete number and unit of measurement
☐	☒	A statement on whether measurements were taken from distinct samples or whether the same sample was measured repeatedly
☐	☒	The statistical test(s) used AND whether they are one- or two-sided<br>*Only common tests should be described solely by name; describe more complex techniques in the Methods section.*
☐	☒	A description of all covariates tested
☐	☒	A description of any assumptions or corrections, such as tests of normality and adjustment for multiple comparisons
☐	☒	A full description of the statistical parameters including central tendency (e.g. means) or other basic estimates (e.g. regression coefficient) AND variation (e.g. standard deviation) or associated estimates of uncertainty (e.g. confidence intervals)
☐	☒	For null hypothesis testing, the test statistic (e.g. F, t, r) with confidence intervals, effect sizes, degrees of freedom and P value noted<br>*Give P values as exact values whenever suitable.*
☒	☐	For Bayesian analysis, information on the choice of priors and Markov chain Monte Carlo settings
☒	☐	For hierarchical and complex designs, identification of the appropriate level for tests and full reporting of outcomes
☐	☒	Estimates of effect sizes (e.g. Cohen's d, Pearson's r), indicating how they were calculated

Our web collection on statistics for biologists contains articles on many of the points above.

Software and code

Policy information about availability of computer code

Data collection	CrowdTangle interface, rtweet (v0.7.0) and academictwitteR (v0.3.1) packages was used for data collection for the news sources Facebook, Twitter and Geolocated Twitter, respectively.
Data analysis	All analyses were performed in R (v4.3.1) and the code is available on OSF. We used lme4 (v1.1-35.1), jtools (v2.2.2), quanteda (v3.3.1), and effectsize (v0.8.6) for analysis and huggingface transformers (v4.30) for classifiers.

For manuscripts utilizing custom algorithms or software that are central to the research but not yet described in published literature, software must be made available to editors and reviewers. We strongly encourage code deposition in a community repository (e.g. GitHub). See the Nature Portfolio guidelines for submitting code & software for further information.

Data

Policy information about availability of data

All manuscripts must include a data availability statement. This statement should provide the following information, where applicable:

- Accession codes, unique identifiers, or web links for publicly available datasets
- A description of any restrictions on data availability
- For clinical datasets or third party data, please ensure that the statement adheres to our policy

The raw social media data are protected and are not available due to data privacy laws. The processed (anonymized, partial) social media data are available on OSF: 10.17605/OSF.IO/RMC3E. Source data are provided with this paper.

Research involving human participants, their data, or biological material

Policy information about studies with human participants or human data. See also policy information about sex, gender (identity/presentation), and sexual orientation and race, ethnicity and racism.

Reporting on sex and gender	Sex and gender are not included in any of the analyses.
Reporting on race, ethnicity, or other socially relevant groupings	NA.
Population characteristics	See above.
Recruitment	See above.
Ethics oversight	This study was approved by the University of Cambridge Research Ethics Committee (PRE.2022.022).

Note that full information on the approval of the study protocol must also be provided in the manuscript.

Field-specific reporting

Please select the one below that is the best fit for your research. If you are not sure, read the appropriate sections before making your selection.

☐ Life sciences ☒ Behavioural & social sciences ☐ Ecological, evolutionary & environmental sciences

For a reference copy of the document with all sections, see nature.com/documents/nr-reporting-summary-flat.pdf

Behavioural & social sciences study design

All studies must disclose on these points even when the disclosure is negative.

Study description	This is a quantitative study. For Studies 1 and 2, we collected Facebook and Twitter posts by pro-Ukrainian and pro-Russian news sources popular in Ukraine, spanning Jul 12, 2021, to Sept 13, 2022. We created context-specific dictionaries of descriptive ingroup and outgroup identity. We used existing language-specific dictionaries of negative and positive affect (Ukrainian LIWC 2015 and Russian LIWC 2007) and translated a moral emotional dictionary from Brady et al. (2017). We first established that before the 2022 Russian invasion of Ukraine, out-group language drove the most engagement on pro-Ukrainian Facebook and Twitter and pro-Russian Facebook. Using sliding window regression analysis, we observed that the influence of identity language dramatically decreased on pro-Ukrainian social media after the invasion. To investigate that further, we created Ukraine-specific dictionaries of ingroup solidarity and out-group hostility and finetuned BERT-NLI models. We observed that ingroup solidarity was the strongest and most consistent predictor of engagement after the invasion and throughout the first six months of the 2022 Russo-Ukrainian war in the news sources data. For Study 3, we used the BERT-NLI models, which we further finetuned, to investigate if solidarity or hostility were likely to get more engagement among a dataset of original, non-reply tweets geolocated to Ukraine by Twitter, posted within the same time window as before and labeled by a RoBERTa classifier as pro-Ukrainian (vs. pro-Russian).
Research sample	For Studies 1 and 2, the research sample included 108 news sources popular in Ukraine (see below) of which 100 had functional Facebook accounts and 93 had Twitter accounts as of August 2021. 15 of the accounts belonged to pro-Russian news sources on both social media platforms. For Study 3, the sample included original, non-reply tweets marked by the academictwitteR package as geolocated to Ukraine. Descriptive statistics for all datasets can be found in Tables S5-14 in the SI.
Sampling strategy	For Studies 1 and 2, we selected the most popular news sources in Ukraine based on the following sources: the Internet Association of Ukraine 100 Most Popular Socio-Political Websites in Ukraine (Dec 2019), the Institute of Mass Information Rating of Ukraine Websites (Aug 2020), Texty.org.ua 50 Most Read Online News Media based on Gemius and TNS rankings, VoxUkraine Media Experts Study, and SimilarWeb Top 15 (Jul-Sept 2021). Links to the above sources and a csv file with news sources and their social media handles can be found on the OSF and in the Supporting Information file. For Study 3, the sample included original, non-reply tweets marked by the academictwitteR package as geolocated to Ukraine.
Data collection	We retrieved Facebook data using the CrowdTangle user interface historic download for each page. We retrieved Twitter data using the R package rtweet, getting the last 3,200 posts for each account on several occasions and removing all duplicates. The geolocated Twitter dataset for Study 3 was collected using the R package academictwitteR with the country setting set to Ukraine.
Timing	Facebook data was collected in bulk using the CrowdTangle historic download for each page in July 11, 2023. The Twitter data was collected on Aug 15, 2021, Jan 18, 2022, Jun 19, 2022 and Sept 14, 2022. The geolocated Twitter dataset for Study 3 was collected on April 13, 2023.
Data exclusions	We could not collect data from 9 (out of 108) news sources on Facebook and 15 on Twitter due to the news source not having a given social media account or the account being blocked by the platform at the time of first data collection (Aug 2021), or issues with the CrowdTangle platform (one pro-Ukrainian news source). We only included the data that spans the same timeline for both social media platforms (Jul 12, 2021 to Sept 13, 2022). All duplicates resulting from the data collection procedure were excluded.
Non-participation	This is an observational study.

Randomization	This is an observational study with no randomization.

Reporting for specific materials, systems and methods

We require information from authors about some types of materials, experimental systems and methods used in many studies. Here, indicate whether each material, system or method listed is relevant to your study. If you are not sure if a list item applies to your research, read the appropriate section before selecting a response.

Materials & experimental systems

n/a	Involved in the study
☒	☐ Antibodies
☒	☐ Eukaryotic cell lines
☒	☐ Palaeontology and archaeology
☒	☐ Animals and other organisms
☒	☐ Clinical data
☒	☐ Dual use research of concern
☒	☐ Plants

Methods

n/a	Involved in the study
☒	☐ ChIP-seq
☒	☐ Flow cytometry
☒	☐ MRI-based neuroimaging

Plants

Seed stocks	NA
Novel plant genotypes	NA
Authentication	NA
